# Reduced IRF4 expression promotes lytic phenotype in Type 2 EBV-infected B cells

**DOI:** 10.1371/journal.ppat.1010453

**Published:** 2022-04-26

**Authors:** Jillian A. Bristol, Joshua Brand, Makoto Ohashi, Mark R. Eichelberg, Alejandro Casco, Scott E. Nelson, Mitchell Hayes, James C. Romero-Masters, Dana C. Baiu, Jenny E. Gumperz, Eric C. Johannsen, Huy Q. Dinh, Shannon C. Kenney

**Affiliations:** 1 Department of Oncology, McArdle Laboratory for Cancer Research, School of Medicine and Public Health, University of Wisconsin-Madison, Madison, Wisconsin, United States of America; 2 Department of Microbiology and Immunology, University of Wisconsin-Madison, Madison, Wisconsin, United States of America; 3 Department of Medicine, School of Medicine and Public Health, University of Wisconsin-Madison, Madison, Wisconsin, United States of America; 4 Department of Biostatistics and Medical Informatics, University of Wisconsin-Madison, Madison, Wisconsin, United States of America; University of Zurich, SWITZERLAND

## Abstract

Humans are infected with two types of EBV (Type 1 (T1) and Type 2 (T2)) that differ substantially in their EBNA2 and EBNA 3A/B/C latency proteins and have different phenotypes in B cells. T1 EBV transforms B cells more efficiently than T2 EBV *in vitro*, and T2 EBV-infected B cells are more lytic. We previously showed that both increased NFATc1/c2 activity, and an NFAT-binding motif within the BZLF1 immediate-early promoter variant (Zp-V3) contained in all T2 strains, contribute to lytic infection in T2 EBV-infected B cells. Here we compare cellular and viral gene expression in early-passage lymphoblastoid cell lines (LCLs) infected with either T1 or T2 EBV strains. Using bulk RNA-seq, we show that T2 LCLs are readily distinguishable from T1 LCLs, with approximately 600 differentially expressed cellular genes. Gene Set Enrichment Analysis (GSEA) suggests that T2 LCLs have increased B-cell receptor (BCR) signaling, NFAT activation, and enhanced expression of epithelial-mesenchymal-transition-associated genes. T2 LCLs also have decreased RNA and protein expression of a cellular gene required for survival of T1 LCLs, IRF4. In addition to its essential role in plasma cell differentiation, IRF4 decreases BCR signaling. Knock-down of IRF4 in a T1 LCL (infected with the Zp-V3-containing Akata strain) induced lytic reactivation whereas over-expression of IRF4 in Burkitt lymphoma cells inhibited both NFATc1 and NFATc2 expression and lytic EBV reactivation. Single-cell RNA-seq confirmed that T2 LCLs have many more lytic cells compared to T1 LCLs and showed that lytically infected cells have both increased NFATc1, and decreased IRF4, compared to latently infected cells. These studies reveal numerous differences in cellular gene expression in B cells infected with T1 versus T2 EBV and suggest that decreased IRF4 contributes to both the latent and lytic phenotypes in cells with T2 EBV.

## Author summary

EBV is an important cause of human B-cell malignancies and transforms B cells *in vitro*. Although there are two different types of EBV (T1 and T2), most research has been performed using T1 strains and much less is known about how T2 EBV interacts with B cells. T2 EBV is most frequently found in regions of Africa where malaria is common, and we recently showed that B cells infected with T2 EBV have more lytic infection compared to B cells infected with T1 EBV. Here we used both bulk and single-cell RNA-seq analysis to globally define the differences in cellular gene expression in early passage EBV-transformed lymphoblastoid cell lines (LCLs) infected with T1 versus T2 EBV strains. These studies reveal that hundreds of cellular genes are differentially expressed in T1 versus T2 LCLs, and that most of these differences in gene expression occur in the latently infected cells. We also find that decreased expression of the cellular transcription factor, IRF4, contributes to the both the latent and lytic phenotypes of T2 EBV infected B cells. Together these results suggest that T1 and T2 EBV have distinctly different effects in infected B cells.

## Introduction

Epstein-Barr virus (EBV) is a cancer-associated gamma herpes virus that infects up to 90% of humans and causes infectious mononucleosis during primary infection. EBV contributes to a variety of different human B-cell malignancies, including Burkitt lymphoma, Hodgkin lymphoma, diffuse large B cell lymphoma and lymphoproliferative disease of immunocompromised patients [1–3]. EBV, like all herpes viruses, can infect cells in either latent or lytic forms. The major reservoir for latent EBV infection in humans is the memory B-cell compartment. In latently infected cells, which can express from 1 to 9 latent viral proteins depending upon the type of viral latency, the virus is replicated once per cell cycle using the EBNA1 protein and the host cell DNA polymerase [2,3]. A form of latent EBV infection referred to as “type III” latency, in which all 9 EBV latent proteins are expressed, is sufficient to transform B cells *in vitro* into long-lived lymphoblastoid cell lines (LCLs) [2,3], and studies examining how various latent EBV proteins collaborate to transform B cells *in vitro* have provided the major model for EBV-induced lymphomas in humans.

The lytic form of EBV infection (in which many viral genes are expressed and the viral genome is replicated using the virally-encoded DNA polymerase) is required for production of infectious virion particles and the spread of the virus from cell-to-cell and host-to-host [3]. Both latent and lytic EBV infection contribute to the development of EBV-associated malignancies, although fully formed tumors are composed primarily of latently infected cells [4]. A variety of previous studies (often performed in Burkitt lymphoma cell lines) have suggested that latently infected B cells can be switched to the lytic form of infection when the B-cell receptor (BCR) is stimulated with antigen and/or cells differentiate into plasma cells [5–10].

There are two different types of EBV (Type 1 (T1) and Type 2 (T2)), but most studies have been performed using T1 EBV strains and hence much less is known about T2 EBV. In western countries, T1 EBV infection is much more common than T2 EBV infection [11,12]. T2 EBV infection most commonly occurs in sub-Saharan Africa and New Guinea, where it is reported to be present in ~25% of the population [11–16]. Humans can be infected with both EBV types simultaneously [16,17], and recombination between T1 and T2 strains is sometimes (albeit rarely) found in sequenced EBV genomes [13].

T1 and T2 EBV strains are most divergent in their EBNA2 and EBNA 3A/B/C latency gene sequences, with the EBNA2 protein sequences having only ~50% identity [13]. EBNA2 is a viral transcription factor that is required for the ability of EBV to transform B cells *in vitro* [3]. T2 EBV strains have a reduced ability to transform B cells *in vitro*, and this defect has been proposed to be largely due to differences in the EBNA2 protein sequences [18–20]. A single amino acid difference (S442D) between the T1 and T2 EBNA2 proteins has been shown to promote T1 EBNA2 binding to EICE motifs, allowing T1 EBNA2 to activate expression of the latent viral LMP1 gene more efficiently in T1 EBV-transformed LCLs [19,20]. LMP1 is an oncoprotein that mimics constitutively active CD40 signaling, and collaborates with EBNA2 to transform B cells *in vitro* [3,21,22]. In addition, differences between T1 and T2 EBNA2 functions also reflect the ability of T2 EBNA2 but not T1 EBNA2 to interact with the cell repressor BS69 [21]. Early passage T2 LCLs express much less LMP1 compared to early passage T1 LCLs, and this difference in LMP1 expression is thought to be the major cause for the reduced transformation ability of T2 EBV strains *in vitro* [20–23]. Nevertheless, this transformation defect may be specific to the formation of *in vitro* LCLs, since T2 EBV strains are not under-represented in human B-cell malignancies (relative to their frequency in the healthy population) and are not deficient for the ability to cause B-cell lymphomas in humanized mouse models [24,25].

We recently discovered that another major phenotypic difference between T1 and T2 EBV-infected B cells is the enhanced ability of B cells with T2 EBV infection to enter the lytic form of viral infection [24]. Apart from the differences in the EBNA2 and EBNA3A/B/C genes, the sequences of T1 and T2 EBV genes are very similar in most other EBV genes, with only a few lytic genes showing consistent differences [12,13]. Importantly, however, essentially all T2 EBV strains contain the Zp-V3 form of the promoter (Zp) driving expression of the BZLF1 immediate-early gene, whereas most T1 strains have the “prototype” form, Zp-P, of the Z promoter [12,13]. The BZLF1 (Z) gene product is a viral transcription factor that cooperates with the EBV BRLF1 (R) IE protein to induce expression of lytic viral proteins, and both Z and R expression are required for the ability of latently infected cells to switch to the lytic form of viral infection [3,5]. The promoters driving Z and R expression are activated by cellular transcription factors. We previously showed that efficient BCR-mediated lytic reactivation in EBV+ B cells requires the cellular NFATc1 transcription factor, and demonstrated that the Zp-V3 form of the BZLF1 promoter (but not the prototype Zp-P form) contains an NFAT binding motif that allows it to be much more strongly activated by BCR stimulation [26]. Furthermore, we recently showed that the higher level of constitutive lytic infection in early passage LCLs with T2 versus T1 EBV infection is due not only to the universal presence of the NFATc1-responsive form of the Z promoter (Zp-V3) in all T2 strains, but also a much higher level of the activated forms of both NFATc1 and NFATc2 in T2 LCLs [24].

In this study, we have used bulk RNA-seq and single-cell RNA-seq (scRNA-seq) to compare the gene expression patterns of T1 EBV- versus T2 EBV- infected LCLs, and to identify the cellular gene expression program associated with the lytically-infected cell population(s). The results of these studies not only confirm that T2 LCLs have many more lytically infected cells than T1 LCLs, but also reveal that both enhanced NFATc1 expression and decreased IRF4 expression in T2 LCLs contribute to lytic reactivation. Furthermore, we find that IRF4 over-expression decreases the levels of both NFATc1 and NFATc2 in EBV-positive Burkitt lymphoma cells and inhibits lytic EBV reactivation in response to BCR activation. Our studies also demonstrate that independent of the effects of lytic EBV infection, the expression levels of nearly 600 cellular genes are significantly different in LCLs infected with T1 versus T2 EBV. Thus, T1 and T2 EBV clearly have different effects in infected B cells.

## Results

### T1 EBV- and T2 EBV- infected lymphoblastoid cell lines have distinctly different cellular gene expression patterns

The effects of T1 EBV versus T2 EBV infection on global cellular gene expression in LCLs has not yet been compared using modern techniques such as bulk RNA-seq and scRNA-seq. Although this type of analysis is essential to define the full scope of differences in the phenotypes of T1 versus T2 EBV-infected B cells, we and others have reported that T2 LCLs become more and more similar to T1 LCLs as they are passaged in culture [19,24], presumably due to selective pressure against cells expressing only low levels of LMP1 and/or undergoing lytic infection. Therefore, in this study we performed bulk RNA-seq using four different early passage T1 LCLs and four different early passage T2 LCLs (cultured for less than 3 months after EBV infection). To ensure that differences observed between T1 and T2 LCLs were not due to strain-specific effects (rather Type-specific effects), we also included two different T1 strains (Mutu and Akata) and two different T2 strains (AG876 and BL5) in this analysis. All LCLs were obtained from the same donor and infected on the same day. Of note, although the T1 Akata strain has the NFATc1-responsive Zp-V3 form of the BZLF1 promoter, we previously showed that LCLs infected with the T1 Akata strain are nevertheless more latent than T2 EBV-infected LCLs since they have less NFATc1 and NFATc2 activity [24].

**Fig 1:**
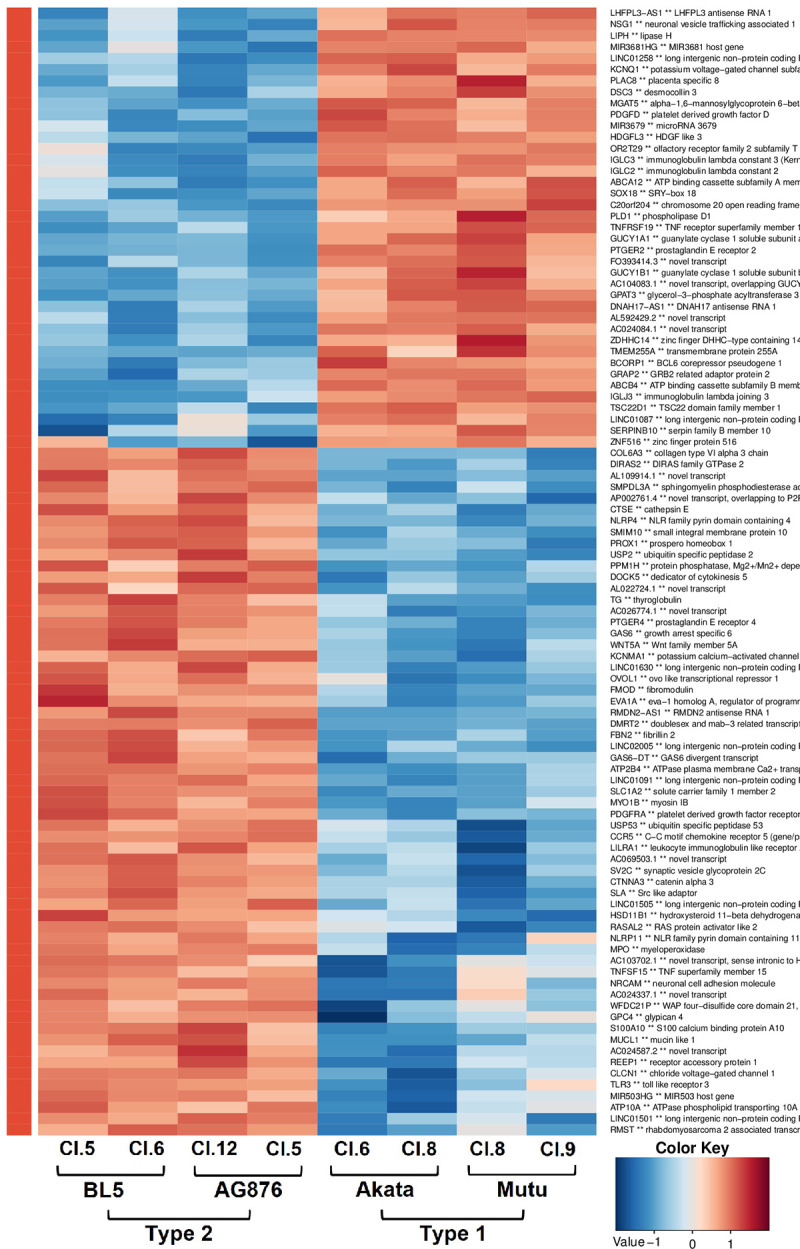
Comparison of cellular transcripts in Type 1 EBV versus Type 2 EBV infected lymphoblastoid cell lines. The top 100 differentially expressed cellular genes in the RNA-seq analysis are shown. Names for each cell line, as well as the EBV type and strain are shown. Red indicates a gene is upregulated in corresponding cells and blue indicates it is down-regulated.

As shown in **Table 1**, cellular gene expression at the bulk level in all four T1 LCLs is remarkably similar, with not a single cellular gene being expressed at a significantly different level in LCLs transformed with the Mutu versus Akata T1 EBV strains. Likewise, cellular gene expression in all four T2 LCLs is also remarkably similar, with no cellular genes expressed at significantly different levels in AG876 versus BL5 infected LCLs. In contrast, cellular gene expression in the T1 versus T2 LCLs is clearly different, with 367 genes being significantly upregulated, and 211 genes being significantly downregulated, in T2 LCLs in comparison to T1 LCLs. **Fig 1** shows a heat map of the top 100 differentially regulated genes in T1 versus T2 LCLs. The expression levels of cellular genes in each specific cell line (and their fold-increase or fold-decrease in T1 versus T2 LCLs) are shown in **S1 Table.** In addition, selected cellular genes of interest that are either up-regulated in T2 LCLs, or down-regulated in T2 LCLs, are presented in **S2 and S3 Tables**, respectively.

**Table 1:** Differentially expressed genes measured in bulk RNA-seq. The number of cellular genes showing at least a two-fold change in gene expression is shown when comparing either the different type 1 EBV strain LCLs (Akata and Mutu), the different type 2 EBV strain LCLs (BL5 and AG876), or type 2 EBV LCLs versus EBV type 1 LCLs.

Comparison	Upregulated	Downregulated
Akata vs. Mutu	0	0
BL5 vs. AG876	0	0
Type 2 vs. Type 1	367	211

Fold change >2, FDR < 0.05

**Fig 2:**
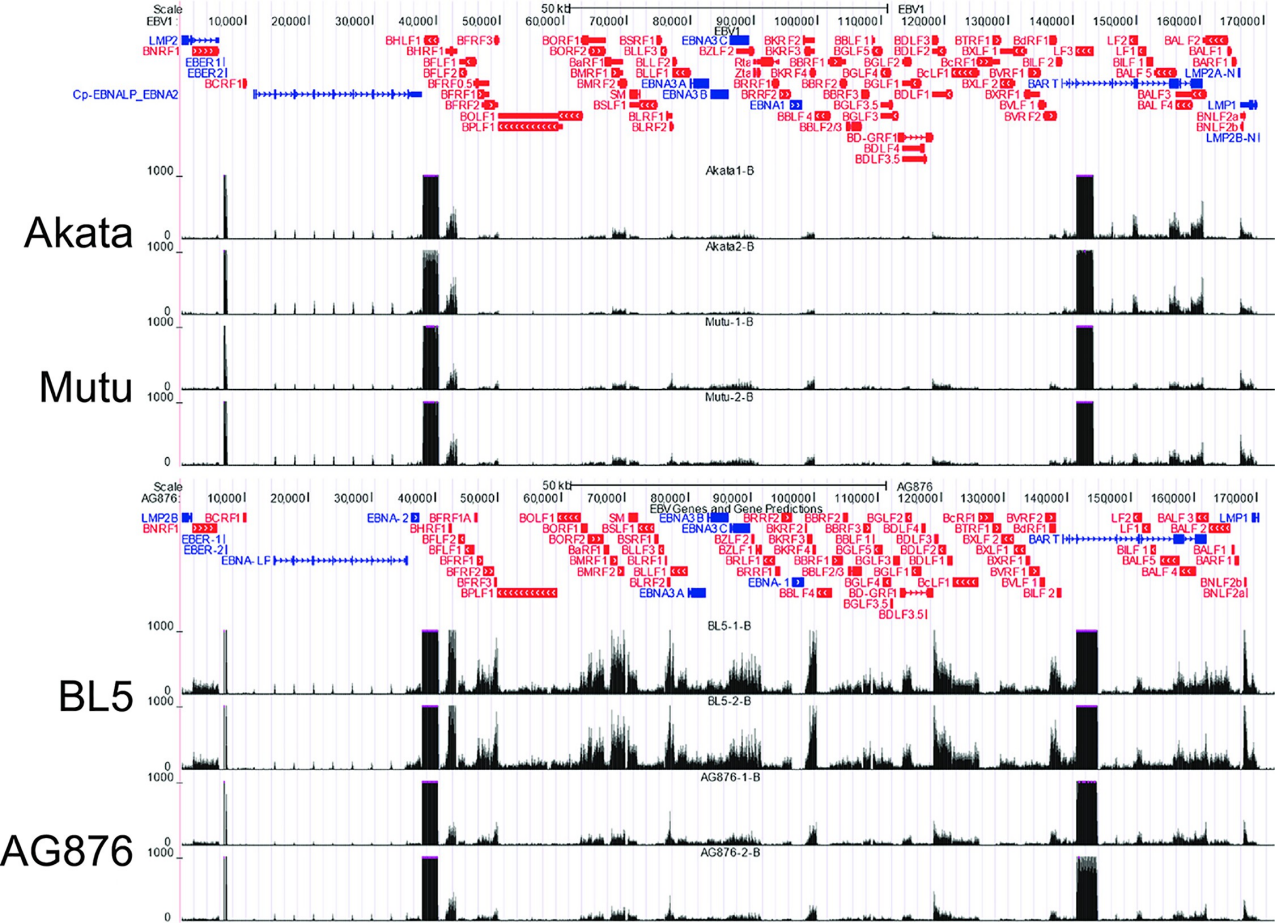
EBV gene expression in T2 virus- versus T1 virus-infected LCLs. RNA-seq reads from LCLs infected with T1 or T2 viruses were aligned to the T1 or T2 EBV genomes, respectively. For each replication, wiggle tracks of normalized read depth are displayed using the UCSC genome browers. Annotation tracks for type 1 and type 2 virues showing latent (blue) genes and lytic (red) genes are displayed above.

The results of the bulk RNA-seq analysis were also used to align viral transcripts to the T1 and T2 EBV genomes, allowing us to compare the levels of different viral transcripts in T1 versus T2 LCLs. As shown in **Fig 2,** this analysis confirmed that T2 LCLs are more lytic than T1 LCLs.

### T2 EBV-infected LCLs have an enhanced signature of BCR stimulation, NFAT activity, and EMT in comparison to T1 EBV-infected LCLs

**Fig 3:**
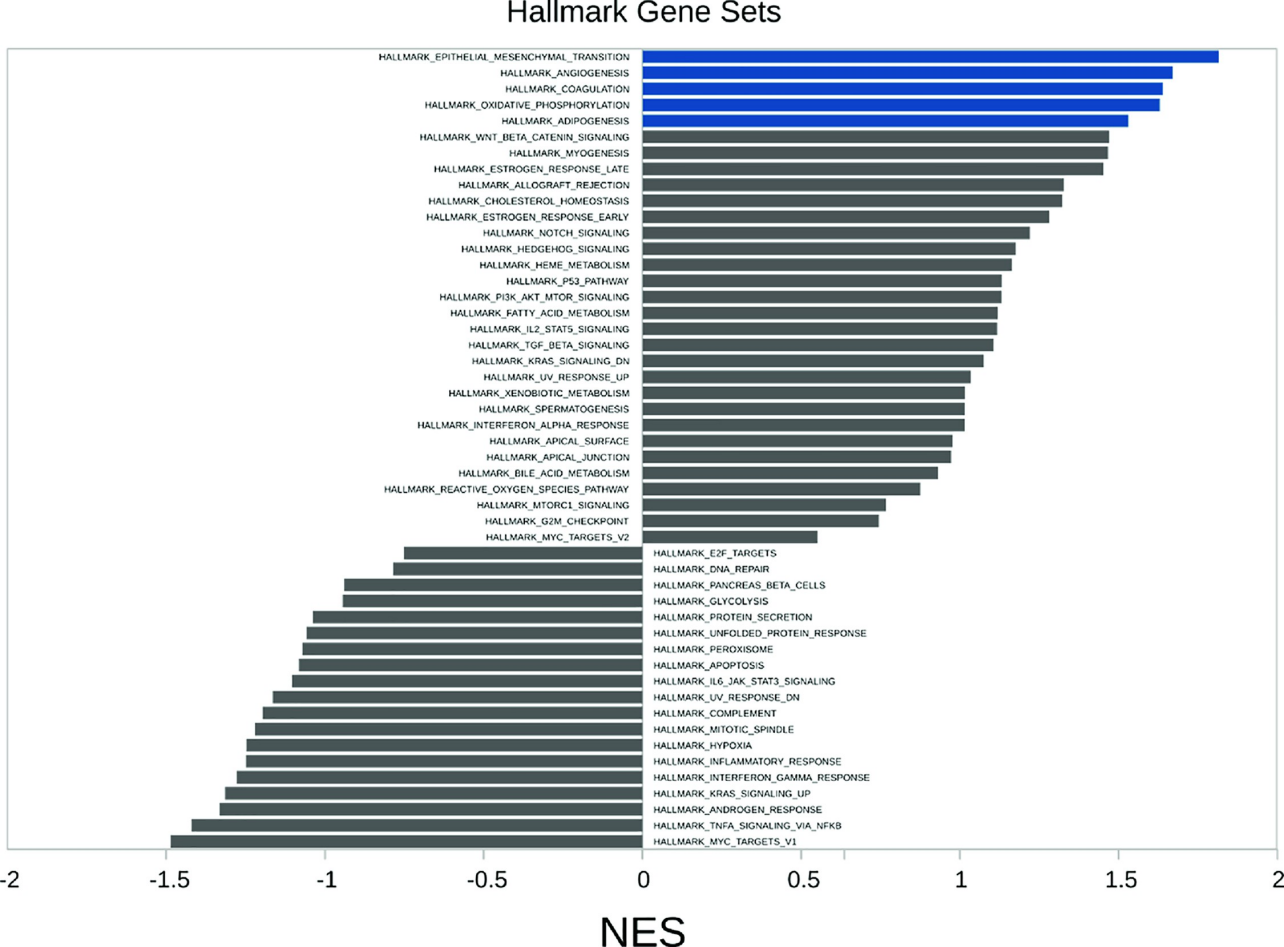
Gene set enrichment analysis (GSEA) using Hallmark gene sets. GSEA comparing T1 versus T2 LCL gene expression was performed using the different Hallmark gene set collection from MSigDB; positive enrichment scores correspond to gene sets enriched in T2 LCL upregulated genes and negative enrichement scores correspond to gene sets enriched in genes upregulated in T1 LCLs. Blue bars are significant with FDR<0.05.

**Fig 4:**
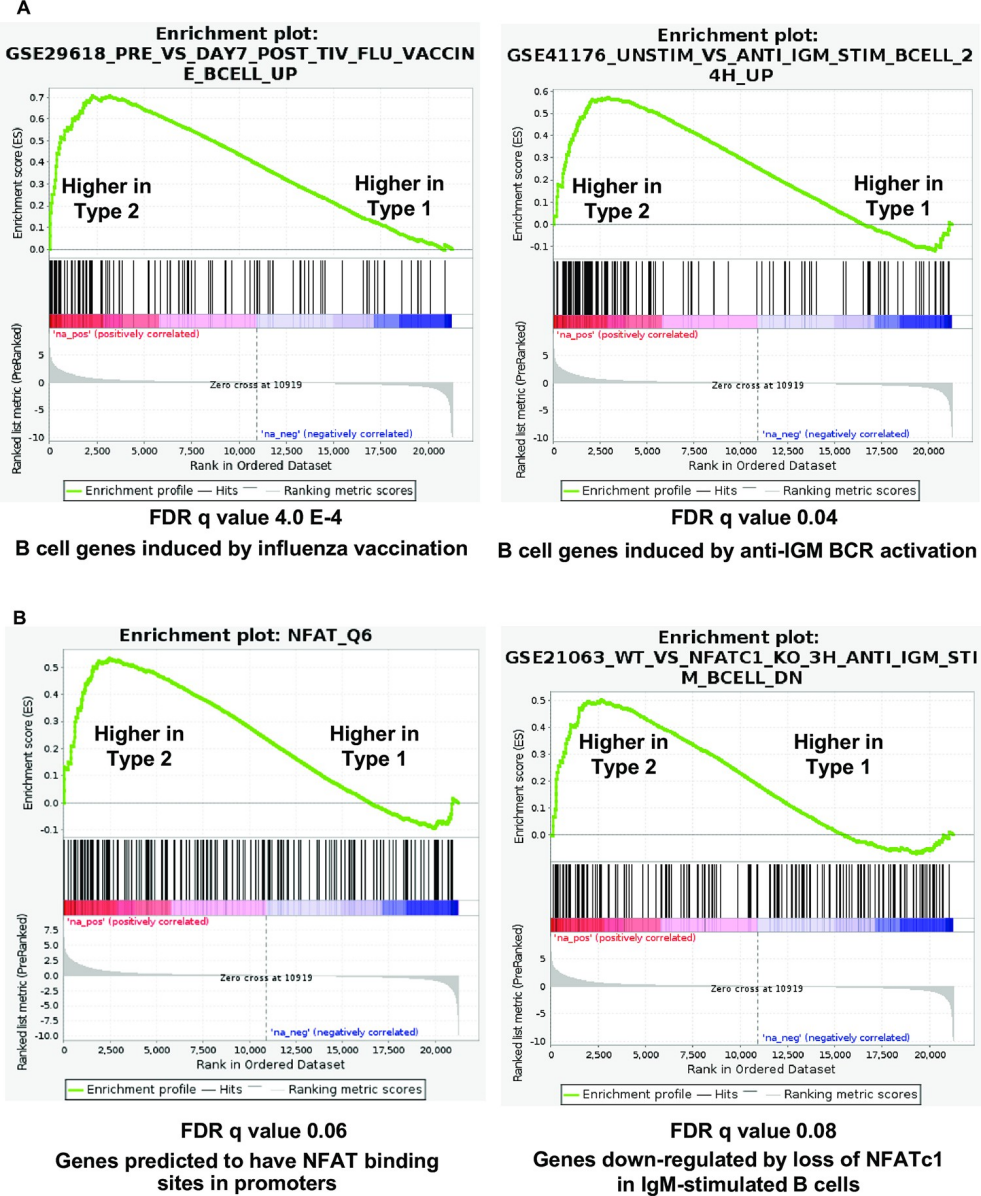
Gene set enrichment analysis (GSEA) suggests increased BCR and NFAT signaling in Type 2 LCLs. **(A)** GSEA plots for the “Pre versus day 7 post TIV flu vaccine B cell up”, and “unstimulated versus anti-IGM stimulated B cells 24 hours up” (**B**). GSEA plots for “NFAT_Q6” and “WT versus NFATC1 KO 3 hour anti-IGM stimulation B cell down”.

To identify signaling pathways differentially regulated by T1 EBV versus T2 EBV infection in LCLs, we analyzed the bulk RNA-seq data using Gene Set Enrichment Analysis (GSEA). GSEA identified that five Hallmark gene sets (epithelial_mesenchymal transition, angiogenesis, coagulation, oxidative phosphorylation and adipogenesis) were significantly enriched in the comparison of gene expression in T2 versus T1 LCLs (**Fig 3)**. As shown in **Fig 4A**, one of the gene sets that was most highly upregulated in T2 LCLs was the “Pre_vs_Day 7_post_TIV_Flu_Vaccine_Bcell_Up” gene set. This set identifies genes upregulated in the B cells of humans injected with influenza vaccine who developed a good antibody response [27], and suggests that T2 LCLs have a signature indicative of antigen-stimulated/BCR-activated B cells. Consistent with this interpretation, another gene set significantly upregulated in T2 EBV LCLs versus T1 LCLs was the “unstimulated versus IGM stimulated B cell 24 hour up” gene set [28].

**Fig 5:**
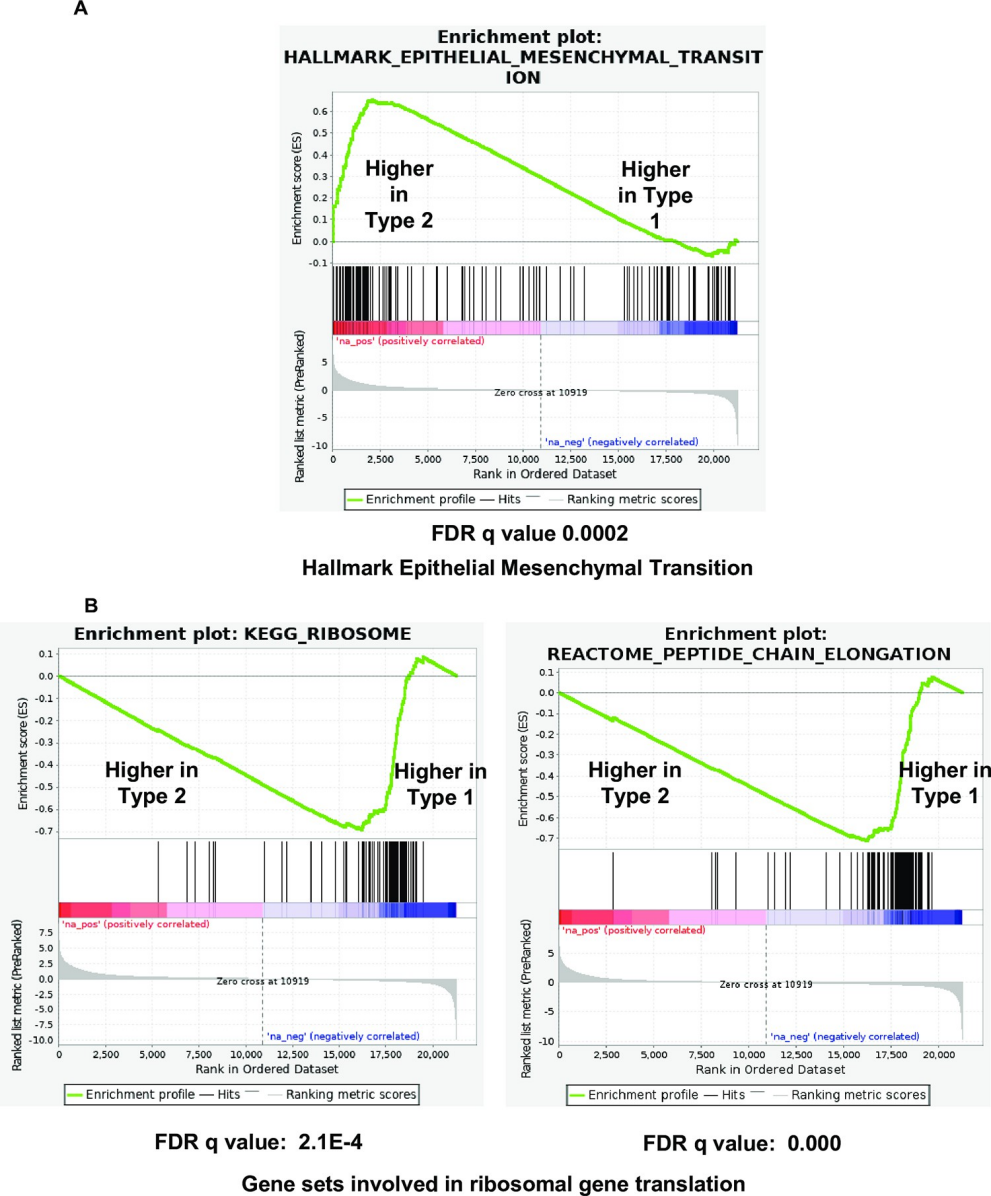
Gene set enrichment analysis (GSEA) suggests increased EMT in Type 2 LCLs. **(A)** GSEA plot for “Hallmark mesenchymal epithelial cell transition.” **(B).** GSEA plots for “KEGG_RIBOSOME” and “REACTOME_PEPTIDE_CHAIN_ELONGATION”.

Since BCR stimulation leads to NFAT activation, and since we previously showed that T2 LCLs have more active (nuclear) NFATc1 and NFATc2 than T1 LCLs [24], we also asked whether gene sets indicative of enhanced NFAT activity are over-represented in T2 LCLs versus T1 LCLs. As shown in **Fig 4B**, T2 LCLs had both upregulated expression of cellular genes predicted to contain NFAT motifs in their promoters (“NFAT_Q6 gene set”) and increased expression of genes in the “Genes down-regulated by loss of NFATc1 in IGM-stimulated B cells” [29], suggesting that T2 LCLs have enhanced NFATc1 and/or NFATc2 activity in comparison to T1 LCLs. In addition, increased expression of genes in the “Hallmark Epithelial Mesenchymal Transition” gene set (**Figs 3 and 5A)** is also consistent with enhanced B-cell activation. Two gene sets that were down-regulated in the T2 LCLs versus T1 LCLs (“KEGG_RIBOSOME” and “REACTOME_PEPTIDE_CHAIN_ELONGATION”) are shown in **Fig 5B**; these gene sets were characterized by genes in the ribosome involved in protein translation.

### NFAT activity contributes to CD11C (ITGAX) upregulation in T2 LCLS

**Fig 6:**
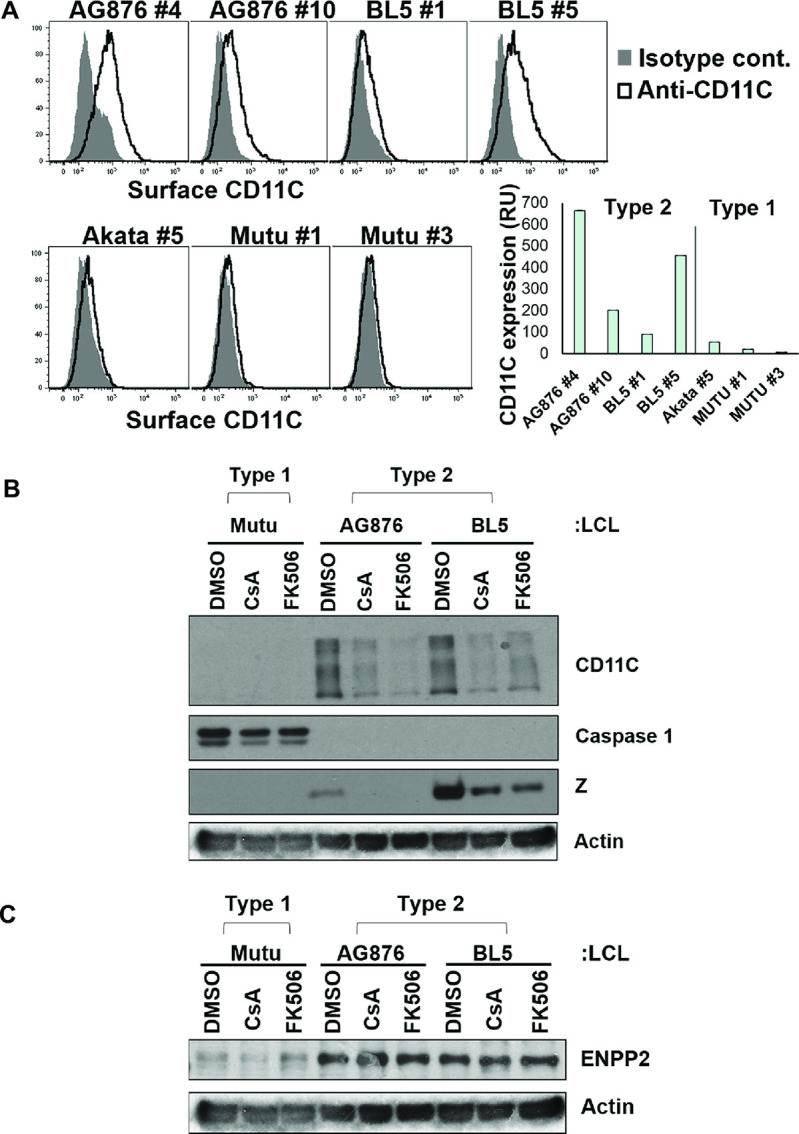
Type 2 LCLs express higher levels of ITGAX (CD11C) and NFAT inhibitors reverse this effect. **A)** The level of surface CD11c expression on various Type 2 LCLs versus Type 1 LCLs was measured by flow cytometry using antibodies directed against CD11C or a control isotype antibody, as indicated. Quantification of the results is also shown (RU, relative units of CD11C mean fluorescence intensity per cell, with isotype control fluorescence subtracted). **B)** T1 or T2 LCLs were treated with NFAT inhibitory drugs, cyclosporin or FK506, or vehicle control for 15 days. Immunoblot was performed to examine expression levels of the CD11C protein, caspase 1, the EBV lytic protein Z, and loading control actin as indicated. **C)** Using the same cell lysates shown in Fig 5B, immunoblot was performed to examine expression of cellular protein ENPP2.

Since the bulk RNA-seq results showed that ITGAX (CD11C) is consistently upregulated in T2 versus T1 LCLs, and CD11C expression has been shown to be activated by BCR stimulation in B cells [30,31], we investigated whether NFAT activity contributes to enhanced CD11C expression in T2 LCLs. FACs analysis of T1 versus T2 LCLs confirmed that T2 LCLs have more surface CD11C expression in comparison to T1 LCLs (**Fig 6A**). To determine if NFATc1/NFATc2 activity contributes to CD11C expression in T2 LCLs, cells were treated with or without the NFAT inhibitors, cyclosporin and FK506, and immunoblot analysis was performed to measure CD11C expression. As shown in **Fig 6B**, T2 LCLs express more CD11C than T1 LCLs on immunoblot, although caspase 1 expression is decreased (consistent with the RNA-seq results shown in **S1 Table**). NFAT inhibitors decreased CD11C expression in T2 LCLs but did not affect caspase 1 expression in T1 LCLs. Expression of ENPP2 (autotaxin), another gene shown to be upregulated in T2 LCLs (**S1 Table**), was also not affected by NFAT inhibitors in T2 LCLs (**Fig 6C**). Thus, increased NFATc1/NFATc2 activity contributes to activation of some, but not all, cellular genes (such as ITGAX) that are upregulated in T2 LCLs.

### T2 LCLs express higher levels of RUNX1, FYN, TNFRSF9, CD9, MHC class II, CYP1B, and AHR in comparison to T1 LCLs

We also confirmed that a number of other cellular genes suggested by the bulk RNA-seq results to be upregulated in T2 LCLs are indeed upregulated at the protein level, including RUNX1, FYN, TNFRSF9 (CD137), CD9, MHC class II, CYP1B, and AHR. The levels of RNA-seq transcript for each gene in various different T1 versus T2 LCLs are shown in **S1 Table** and the protein levels are shown in **S1 Fig**. Of note, the confirmatory western blot experiments were often performed using independently generated (different) EBV lines than those used to obtain the original RNA-seq results. Although the protein results for each gene are largely compatible with the RNA-seq results, the occasional differences observed in certain T1 or T2 lines likely reflects both the difficulty in obtaining adequate protein from very early passage LCLs (particularly T2 LCLs) to perform the various different western blot analyses shown, as well as the selection pressure for T2 LCLs to become more similar to T1 LCLs as they are continually passaged in culture.

Expression of RUNX1 (which is clearly expressed at higher levels at both the RNA and protein levels in early passage T2 versus T1 LCLs) (**S1 Table** and **S1A Fig**), has been previously reported to be incompatible with the long-term survival of T1 LCLs [32]. In LCLs, induction of RUNX3 by EBNA2 represses RUNX1 transcription [32], although we found that RUNX3 levels are similar in T1 and T2 LCLs (**S1A Fig**). Thus, differences in T1 versus T2 EBNA2 protein functions may lead to increased RUNX1 expression in T2 LCLs. Another gene generally upregulated at both the RNA and protein levels in T2 LCLs is FYN **(S1 Table and S1B Fig**). The SRC kinase FYN is involved in mediating early components of the BCR signaling pathway [33], suggesting that increased FYN expression in T2 LCLs could contribute to their enhanced NFAT activity. Another gene whose expression is induced at both the RNA and protein level in T2 LCLs is TNFRSF9 (CD137) (**S1 Table** and **S1C Fig)**. CD137 expression is known to be induced by BCR signaling, although it can also be activated by LMP1 [34,35]. Since T2 LCLs have less LMP1 expression compared to T1 LCLs [24], this result is consistent with increased BCR signaling in T2 LCLs. The CD9 gene is also generally over-expressed at both the RNA and protein levels in T2 LCLs 9 (**S1 Table** and **S1D Fig**). CD9 is a tetraspanin that is reported to be over-expressed in B-reg (immunosuppressive) B cells [36,37]. Interestingly, T2 LCLs, like B-regs, express a higher level of the immunosuppressive IL-10 cytokine (**S1 and S2 Tables)** in the RNA-seq analysis. However, T2 LCLs also generally express a higher level of MHC class II (HLA-DR) at both the RNA-seq and protein levels (**S1 Table** and **S1E Fig**), consistent with their highly activated state but also suggesting that they might present EBV antigens more efficiently on MHC class II.

T2 LCLs also express more of both the AHR receptor, and the AHR-induced target, CYP1B, in comparison to T1 LCLs (**S1 Table** and **S1F Fig**). BCR signaling has been shown to induce AHR activation, and AHR signaling is thought to inhibit BCR signaling [38]. To determine if AHR signaling might be contributing to increased lytic infection in T2 EBV infected LCLs, cells were treated with or without an AHR activating compound, L-Kyn, and the amount of lytic EBV protein expressed was then assessed by immunoblot. Although we found that the AHR activating compound increased CYP1B expression as expected, it actually decreased the amount of lytic protein expression (**S2 Fig**). Thus, BCR activation of the AHR pathway may act as a negative feedback mechanism to prevent excessive lytic EBV infection in T2 EBV-infected B cells.

### T2 LCLs have decreased IRF4, EBF1 and caspase 1 expression compared to T1 LCLs

**Fig 7:**
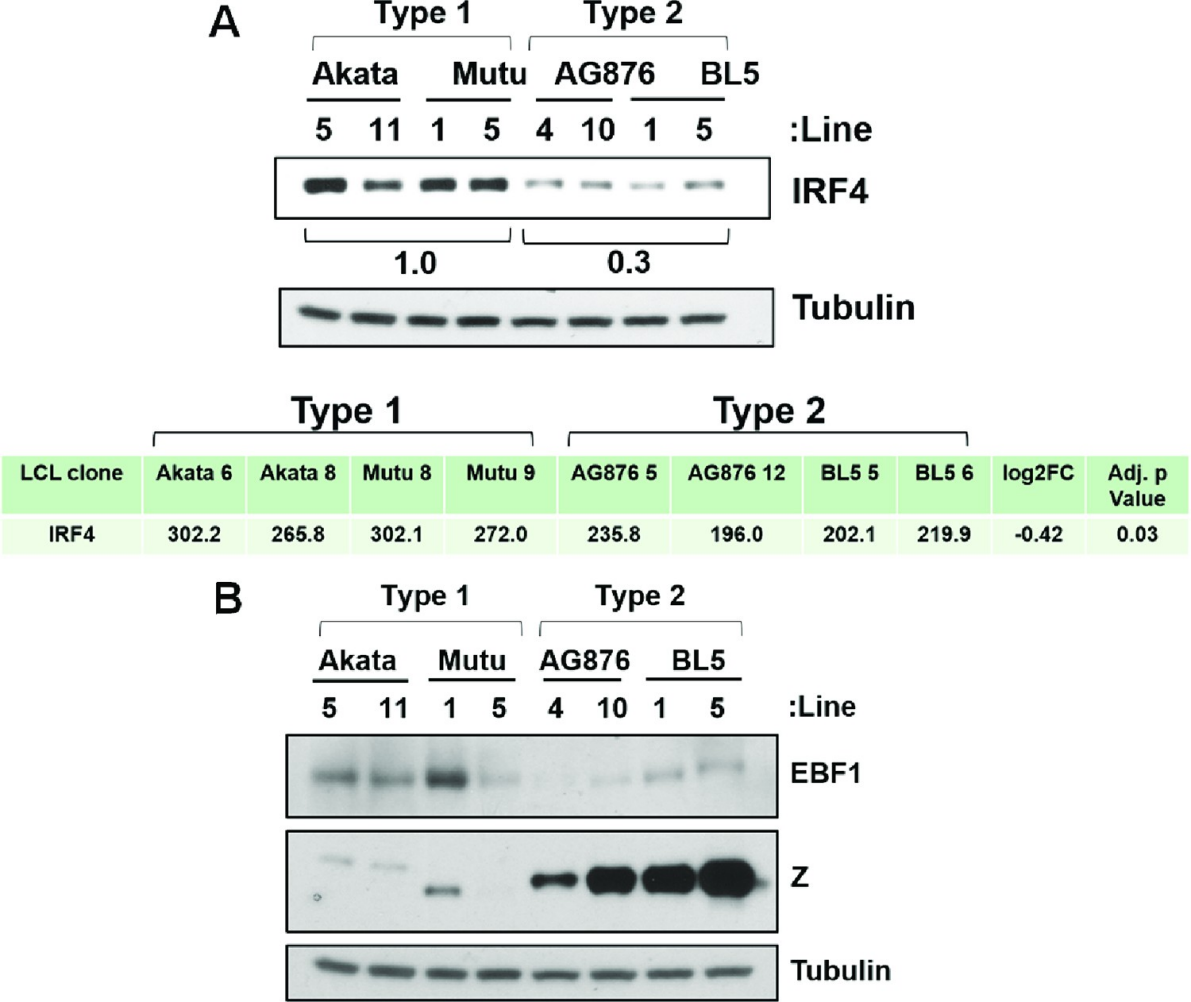
T2 LCLs express lower levels of IRF4 and EBF1 compared T1 LCLs. Immunoblot analyses were performed to compare the protein expression levels of IRF4 **(A)** or EBF1 and Z **(B)** in T1 versus T2 LCLs as indicated (using the same cell lysates). Tubulin was used as loading control. The numbers below the IRF4 immunoblot quantify the results using Image Studio Lite software to normalize the level of IRF4 expression to tubulin expression. Results are presented as the ratio of IRF4 expression relative to tubulin, in T2 cells (averaged) relative to T1 cells (averaged). The T1 IRF4 value is set as 1. The transcript expression of IRF4 (as determined by RNA-seq) is also shown for various different Type 1 and Type 2 LCL lines, along with the log2FC fold change in gene expression in T2 versus T1 cell lines, and the adjusted p value.

In addition to the numerous cellular genes that have increased expression in T2 LCLs, hundreds of other potentially interesting and important cellular genes are down-regulated in T2 versus T1 LCLs (some of which are shown in **S3 Table).** Amongst these, we chose to validate IRF4, EBF1 and caspase 1 at the protein level. We confirmed that T1 LCLs have increased expression of IRF4 (**Fig 7A**), EBF1 **(Fig 7B**) and caspase 1 (**Fig 6B**) at both the RNA transcript (**S1 Table**) and protein (**Figs 6B and 7**) levels. Interestingly, the decrease in IRF4 expression in T2 versus T1 LCLs is even more striking at the protein level in comparison to the RNA level, suggesting that its expression may also be downregulated in T2 LCLs via post-transcriptional mechanism(s).

### T2 LCLs share similarities with the gene expression program of IRF4-KO Type 1 LCLs

**Fig 8:**
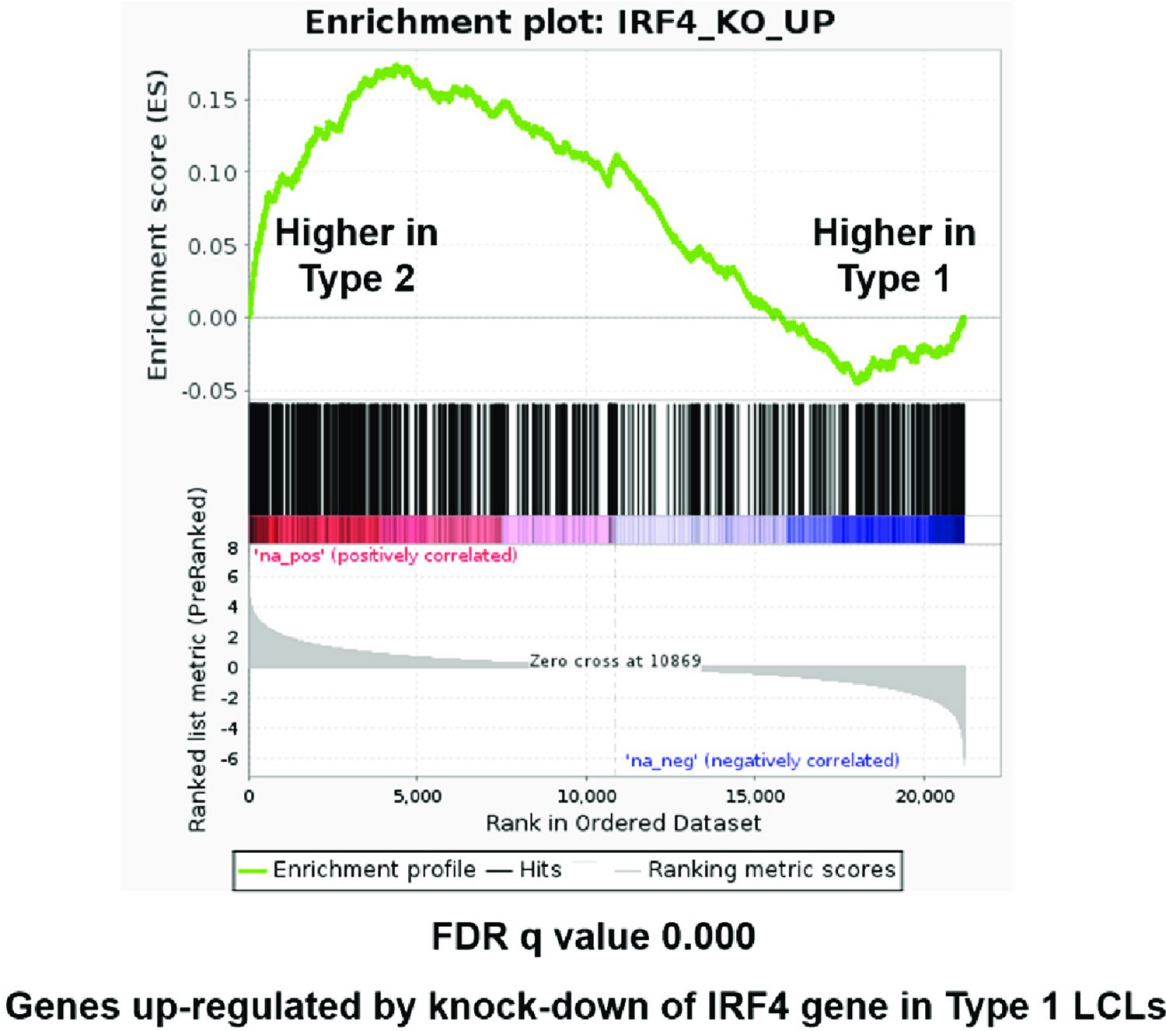
Genes upregulated by IRF4 knockdown in Type 1 LCLs are enriched in unedited Type 2 versus Type 1 LCLs. Gene Set Enrichment Analysis (GSEA) was performed on the bulk Type 2 versus Type 1 LCLs using a gene set constructed from previously published findings of genes upregulated in Type 1 LCLs targeted by IRF4 sgRNAs compared to control sgRNAs by CRISPR/Cas9 [39].

We considered the possibility that decreased IRF4 expression contributes to the lytic phenotype of T2 EBV-infected LCLs since IRF4 (although essential for T1 LCL survival [39,40]) has been shown to inhibit BCR signaling [41], and decreased IRF4 expression enhances the growth of BCR-dependent chronic lymphocytic leukemia tumors in both humans and mouse models [42–45]. To assess whether the gene expression differences in T2 LCLs versus T1 LCLs could at least partially reflect decreased IRF4 expression in the T2 LCLs, we used GSEA to compare cellular gene expression in our bulk RNA-seq analysis of T2 versus T1 LCLs, with a previously published list of cellular genes upregulated in IRF4 knock-out versus control T1 LCLs [39]. This analysis revealed the genes upregulated in IRF4-knockout LCLs are enriched in the T2 LCLs compared to the T1 LCLs (**Figs 8** and **S3**). Some of cellular genes of particular interest that are similarly activated in Type 2 versus Type 1 LCLs, or when IRF4 is knocked-out in Type 1 LCLs, are shown in **S4 Table**. Most notably, knock-out of IRF4 in Type 1 LCLs induces both NFATc1 and NFATc2 expression, as well as expression of the BCR-responsive genes, ITGAX (CD11C) and TNFRSF9 (**S4 Table).** Expression of the FYN and CD9 genes is also increased in the IRF4 knock-out T1 LCLs. These results suggest that decreased IRF4 expression in the Type 2 LCLs is at least partially driving the differences in T2 versus T1 cellular gene expression pattern in our experiments.

### IRF4 negatively regulates lytic EBV Reactivation in T1 LCLs

**Fig 9:**
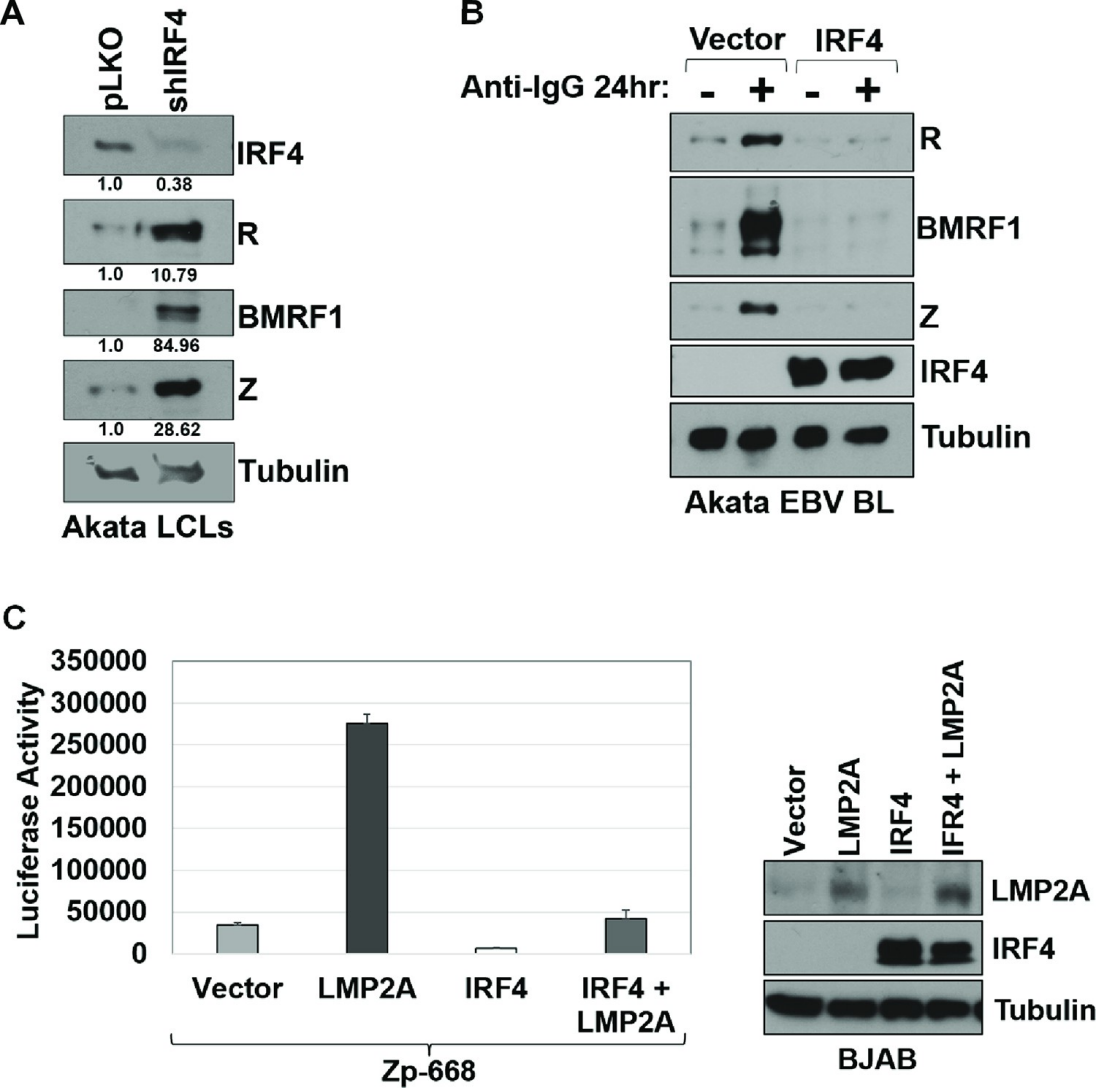
IRF4 suppresses lytic EBV reactivation. **(A)** Akata LCLs were infected with lentiviruses expressing IRF4 targeted shRNAs or a control shRNA vector, selected with puromycin for 7 days, and then immunoblot analysis was performed to detect expression of IRF4, the lytic viral proteins BZLF1 (Z), BRLF1 (R), BMRF1, or tubulin as indicated. The numbers below each immunoblot quantify the results using Image Studio Lite software to normalize the levels of IRF4, R, BMRF1, and Z expression to tubulin expression. Results are presented as the ratio of IRF4, R, BMRF1, and Z expression relative to tubulin in shIRF4 cells relative to vector control (pLKO) cells. Vector control values are set as 1. **(B)** EBV positive Akata Burkitt lymphoma cells were stably infected with pLKO (vector control) or IRF4 lentiviruses. Both Akata BL lines were treated with or without anti-IgG for 24 hours to induce BCR signaling, and then harvested for immunoblot to detect expression of the lytic viral proteins Z, R, and BMRF1, IRF4, and tubulin loading control as indicated. **(C)** EBV negative BJAB Burkitt cells were nucleofected with a full-length Z promoter-luciferase construct and either vector control, an LMP2A expression vector, an IRF4 expression vector, or both LMP2A and IRF4 expression vectors and luciferase assays were performed 2 days later. The experiment was performed in triplicate and repeated twice with similar results. The amount of luciferase activity produced by each condition in one experiment is shown in the left panel. Immunoblot analysis of the LMP2A, IRF4, and tubulin proteins in each condition shown in the luciferase assay is shown in the right panel.

To determine if increased IRF4 expression in T1 LCLs might be contributing to their more latent phenotype, T1 LCLs infected with the Akata EBV strain were infected with lentivirus expressing shRNAs directed against the IRF4 gene, or lentivirus expressing a control shRNA, and then selected for 7 days in culture with puromycin as described in the Materials and Methods. T1 Akata LCLs were chosen for these studies since the Akata strain contains the Zp-V3 form of the BZLF1 promoter and hence can respond more strongly to stimuli mediated via increased BCR signaling. Long-term complete knock-out of IRF4 is not possible in LCLs, as IRF4 is essential for LCL survival [39,40]. Immunoblot analysis was performed to assess knock-down of IRF4, as well as lytic EBV protein expression. As shown in **Fig 9A**, knock-down of IRF4 expression in Akata LCLs increased expression of the viral lytic proteins, BZLF1, BRLF1 and BMRF1. Similar results were obtained using two different IRF4 shRNA combinations (**S4 Fig**). Conversely, over-expression of IRF4 in the EBV-positive Akata Burkitt lymphoma line (which normally expresses no detectable IRF4 protein) decreased constitutive (and BCR-activated) expression of the BZLF1, BRLF1 and BMRF1 lytic proteins **(Fig 9B)**. These results reveal that IRF4 levels play a critical role in modulating the level of lytic EBV infection in LCLs. To further investigate potential mechanisms by which IRF4 inhibits lytic EBV reactivation in B cells, we examined the effect of IRF4 on the promoter activity of the Zp-V3 form of Zp using a luciferase reporter gene assay. Co-transfection of an IRF4 expression vector with the Zp-V3 luciferase vector in EBV-negative BJAB Burkitt lymphoma cells decreased both the constitutive activity, and LMP2A-mediated activation, of the Zp-V3 promoter (**Fig 9C**).

**Fig 10:**
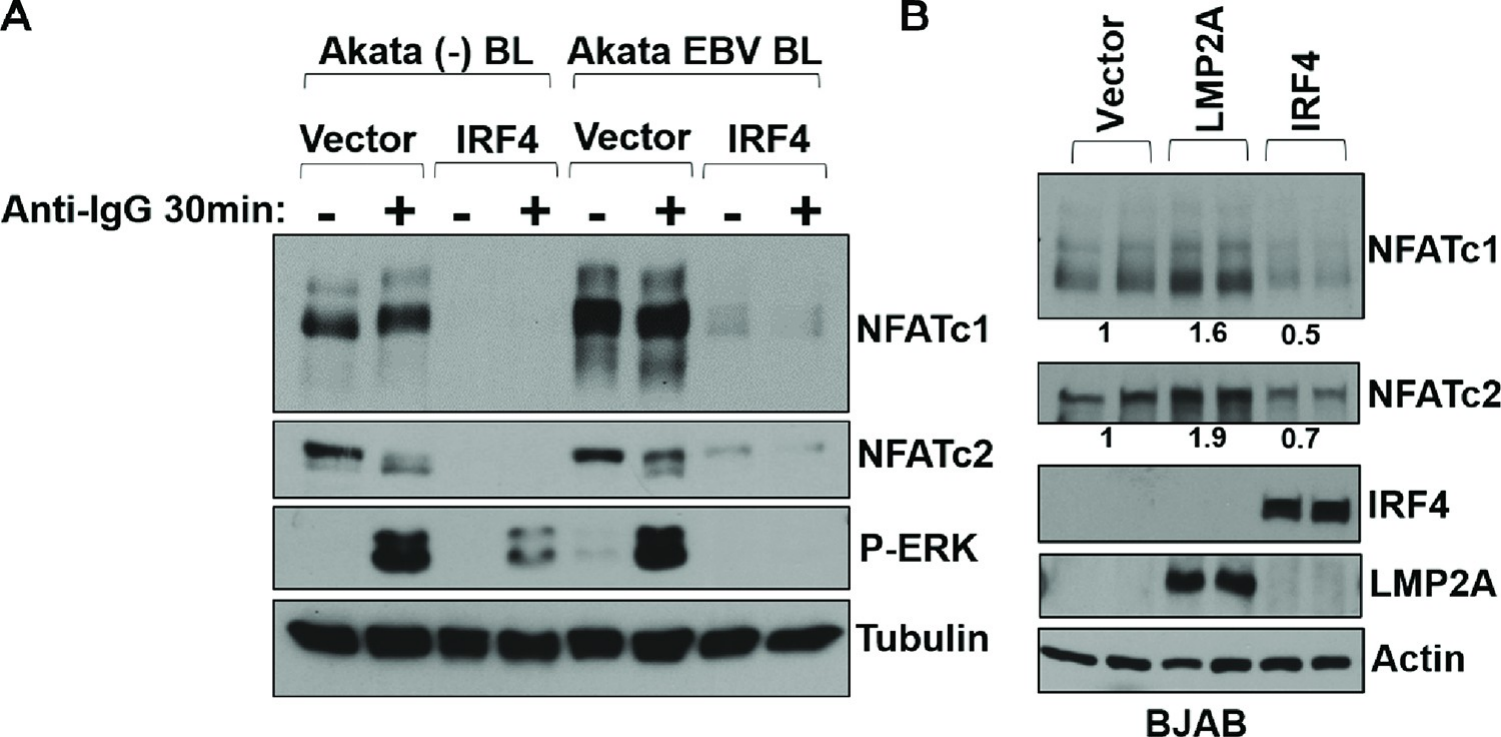
IRF4 inhibits NFATc1 and NFATc2 expression. **(A)** EBV positive or negative Akata BLs stably infected with pLKO (vector control) or IRF4 lentiviruses were treated with or without anti-IgG for 30 minutes and then harvested for immunoblot to detect expression of NFATc1, NFATc2, phospho-ERK, and tubulin loading control as indicated. **(B**) EBV-negative BJAB B cells were nucleofected with vector control, an LMP2A expression vector or an IRF4 expression vector as indicated then harvested after 48 hours for immunoblot analysis of the NFATc1, NFATc2, IRF4, LMP2A, and actin proteins. The numbers below the NFATc1 and NFATc2 immunoblots quantify the results using Image Studio Lite software to normalize the levels of NFATc1 and NFATc2 expression to actin expression. Results are presented as the ratio of NFATc1 and NFATc2 expression relative to actin for each condition, then averaged for each duplicate, and divided by the vector control values. Vector control values are set as 1.

Since we previously showed that the ability of the EBV-encoded LMP2A protein (as well as BCR signaling) to activate the Zp-V3 promoter requires NFAT activity and the promoter NFAT binding site [26], we next determined whether IRF4 over-expression in EBV-negative BJAB Burkitt cells, EBV-negative Akata Burkitt cells, or EBV-positive Akata Burkitt cells affects expression of either the NFATc1 or NFATc2 proteins. As shown in **Fig 10A and 10B**, over-expression of IRF4 decreased expression of both the NFATc1 and NFATc2 proteins in BJAB and Akata BL lines. Consistent with its previously reported ability to inhibit BCR-mediated signaling [41], IRF4 expression also decreased the effect of BCR activation on phospho-ERK induction. Together, these results suggest that IRF4 at least partially decreases lytic EBV reactivation by decreasing NFAT activity in B cells, as well as other downstream mediators of BCR signaling.

### EBF1 negatively regulates the activity of promoters for the IE BZLF1 and BRLF1 genes

Since increased EBF1 expression was previously shown to be highly inversely correlated with enhanced lytic viral infection in a series of T1 LCLs [46], and EBF1 was recently shown to inhibit the activity of the BRLF1 IE promoter in epithelial cells [47], we hypothesized that decreased expression of EBF1 in T2 EBV-infected LCLs also contributes to their lytic phenotype. As we were unable to achieve sufficient knock-down of EBF1 to examine this possibility in LCLs (perhaps reflecting the fact that EBF1 is required for the survival of these cells) [48], we performed reporter gene assays in the EBV negative AGS gastric carcinoma cell line to examine the effect of co-transfected EBF1 protein on the activity of the EBV BZLF1 (Zp) or BRLF1 (Rp) IE promoters (**S5 Fig**). We confirmed that EBF1 inhibits activity of the BRLF1 promoter and found that it also inhibits the activity of the BZLF1 promoter. Thus, it is likely that decreased levels of EBF1 in T2 LCLs also contribute to their more lytic phenotype.

### Single-cell RNA-seq analysis reveals increased NFATc1 and decreased IRF4 expression in lytically infected cells

**Fig 11:**
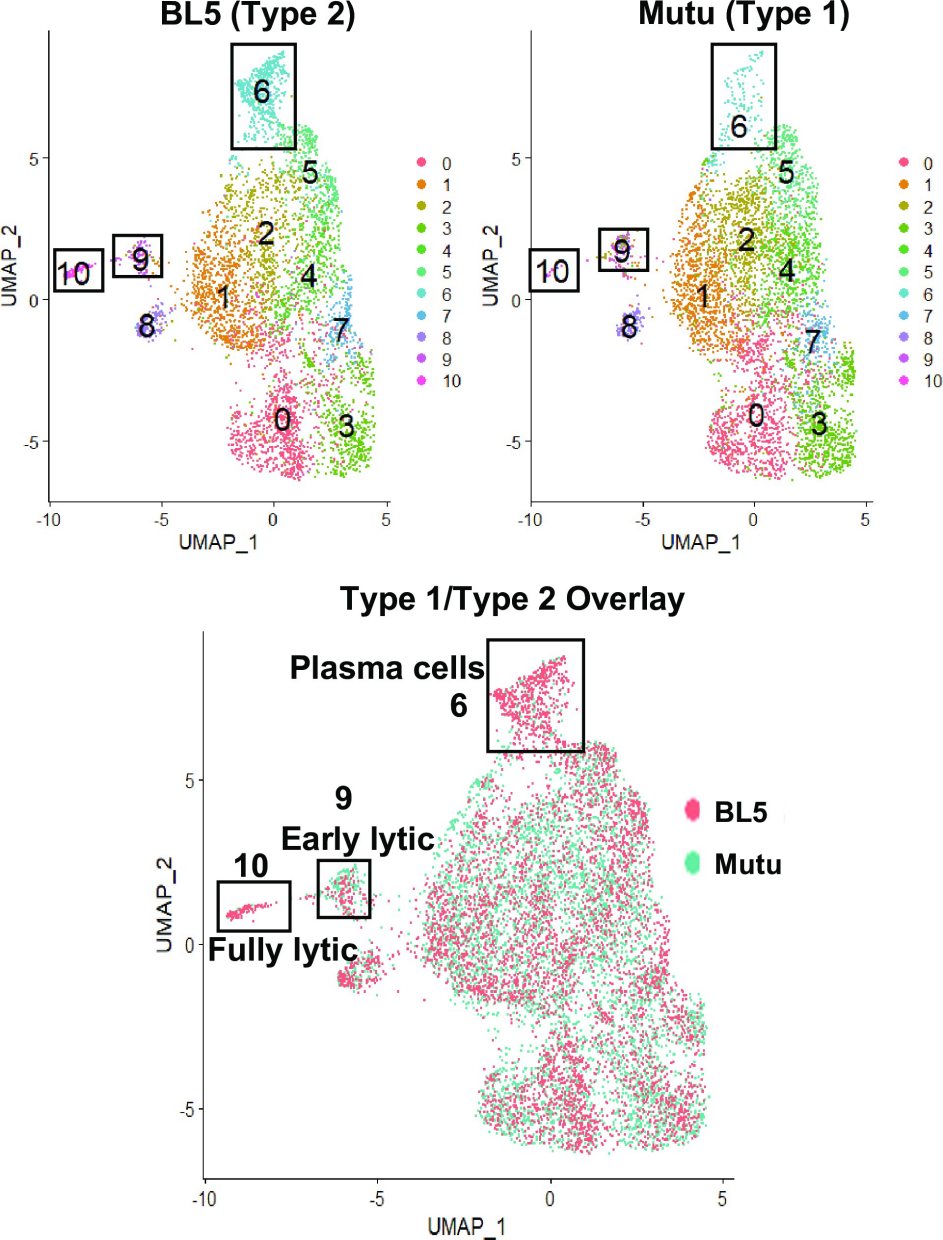
Both T1 and T2 LCLs can be separated into 11 separate clusters in scRNA-seq analysis. Single cell projection onto 2 UMAP dimensions using high dimensional reduction (see Materials and Methods) separated by BL5 and Mutu samples (top) as well as together (bottom) with overlaid different colors are shown.

**Fig 12:**
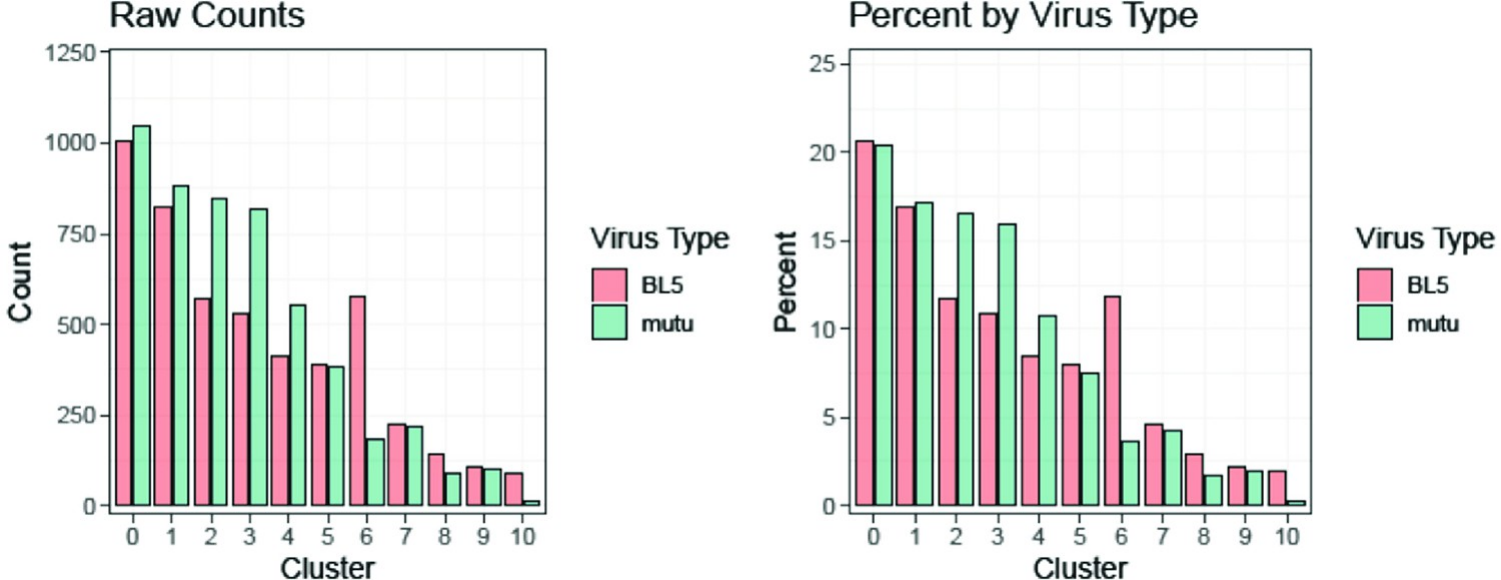
Both T1 and T2 LCLs can be separated into 11 separate clusters in scRNA-seq analysis. 4,879 BL5 and 5,124 Mutu cells analyzed after QC filtering (see Materials and Methods) were distributed across 11 clusters; frequencies of 11 clusters in Mutu and BL5 samples are shown.

To compare gene expression at the single-cell level, we performed scRNA-seq of a Type 1 LCL (Mutu strain) versus a Type 2 LCL (BL5 strain). Integration of two datasets based on canonical correlation analysis followed by graph-based clustering analysis [49] revealed 11 clusters from 4,879 BL5 LCL and 5,124 Mutu LCL single cells (**Fig 11**) after QC and filtering low quality cells (see Materials and Methods). The majority of these clusters (clusters 1,2,3,4,5,7, and 8) contained latently infected cells and had less than 2-fold changes in the numbers of T1 versus T2 LCLs **(Fig 12).**

**Fig 13:**
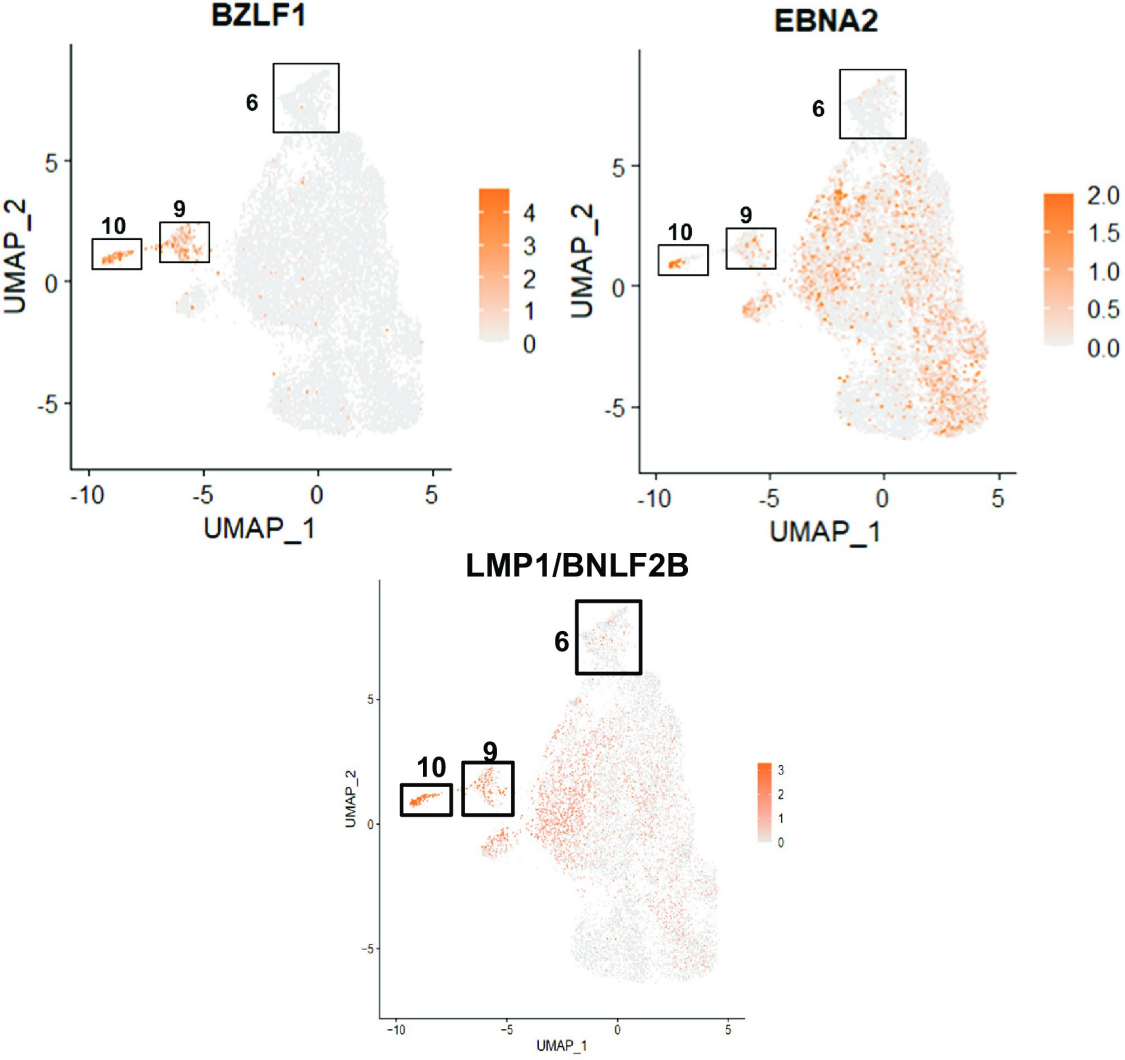
BZLF1 (lytic gene expression) is limited to clusters 9 and 10, whereas latent proteins EBNA2 and LMP1 are expressed more broadly. Expression values are based on log normalized counts. LMP1/BNFL2B expression (overlapping transcripts) was log transformed and averaged to show grouped expression and overcome 3’ prime bias with LMP1.

**Fig 14:**
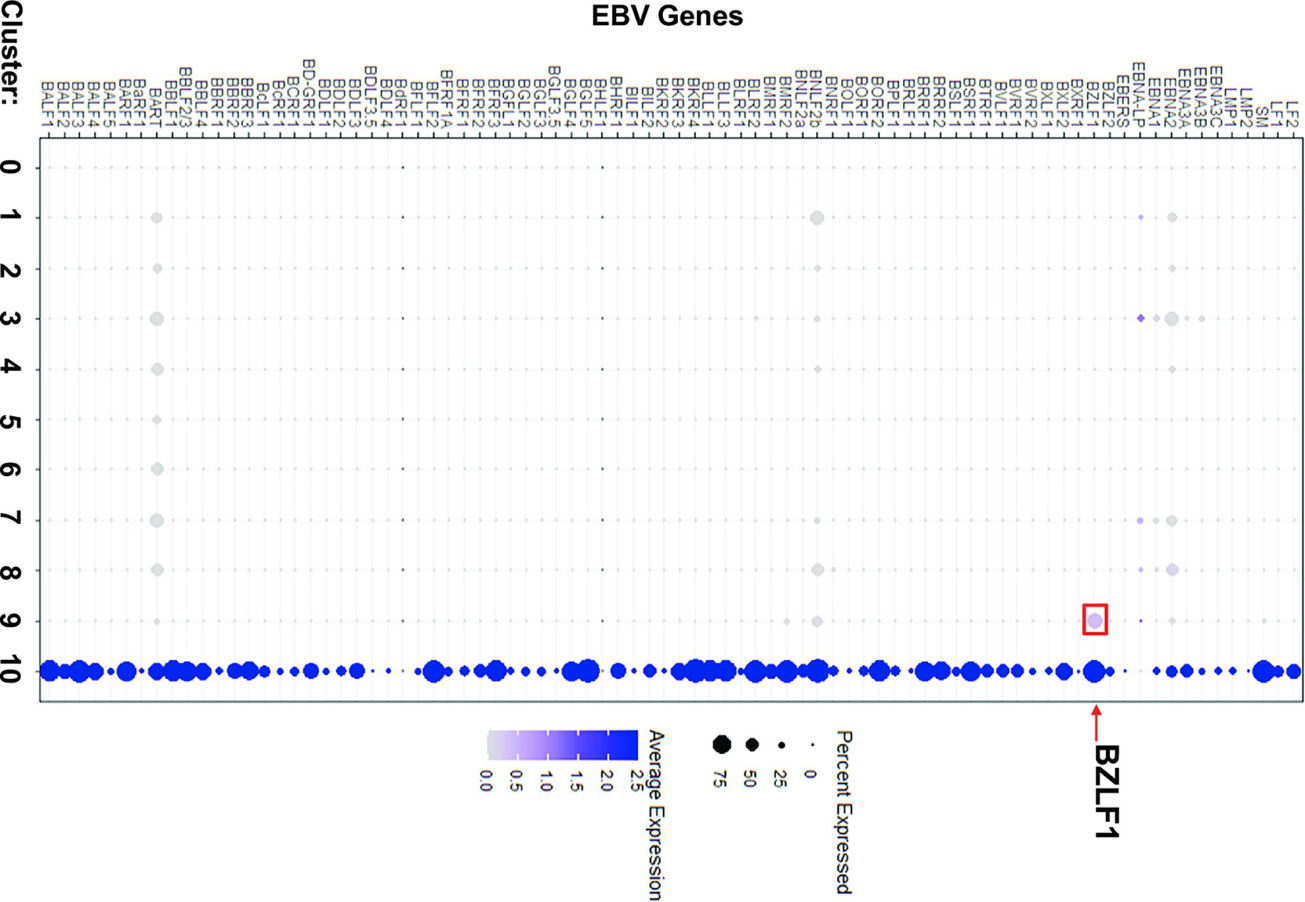
Cluster 10 is much more lytic than cluster 9. A dot plot representing each of the EBV specific genes within the single cell analysis demonstrates lytic activation by scaled expression.

**Fig 15:**
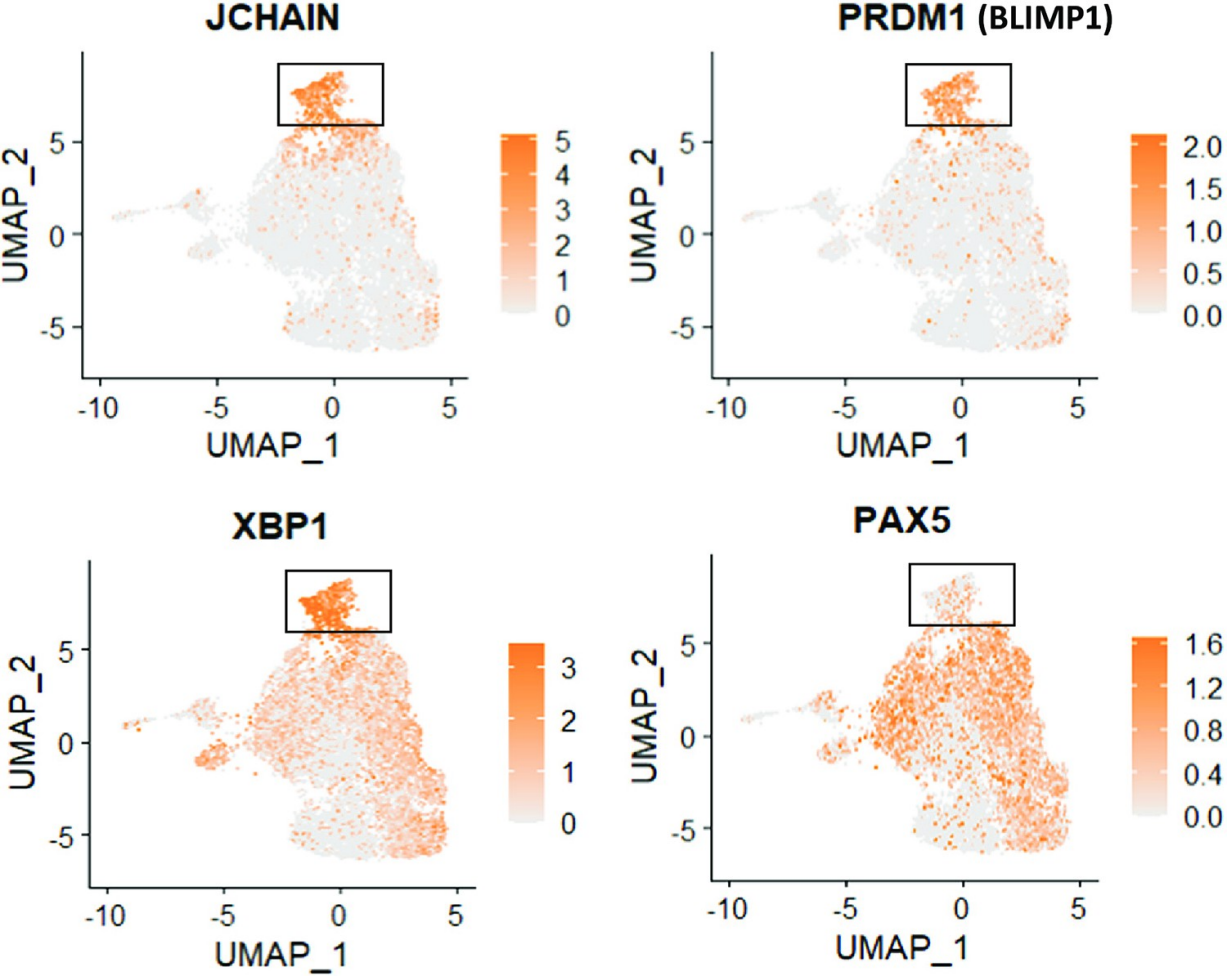
Gene expression analyses of Cluster 6. Cluster 6, which was found to be enriched within the type 2 sample (**Figs 11 and 12**), has a gene expression signature of plasmablast-like cells (J chain, PRDM1, and XBP1 positive, and PAX5 negative).

However, two of the clusters (cluster 6 and cluster 10) contained many more T2 LCLs than T1 LCLs **(Fig 12)**. Cluster 10 cells had fully lytic EBV infection, since cells in this cluster had a high level of all EBV transcripts (**Fig 13 and 14**). T2 LCLs were also over-represented in Cluster 6. This cluster consists of plasmablast-like cells, since it has high level of expression of the plasma cell markers PRDM1 (BLIMP1), XBP1 and J chain (**Fig 15**).

**Fig 16:**
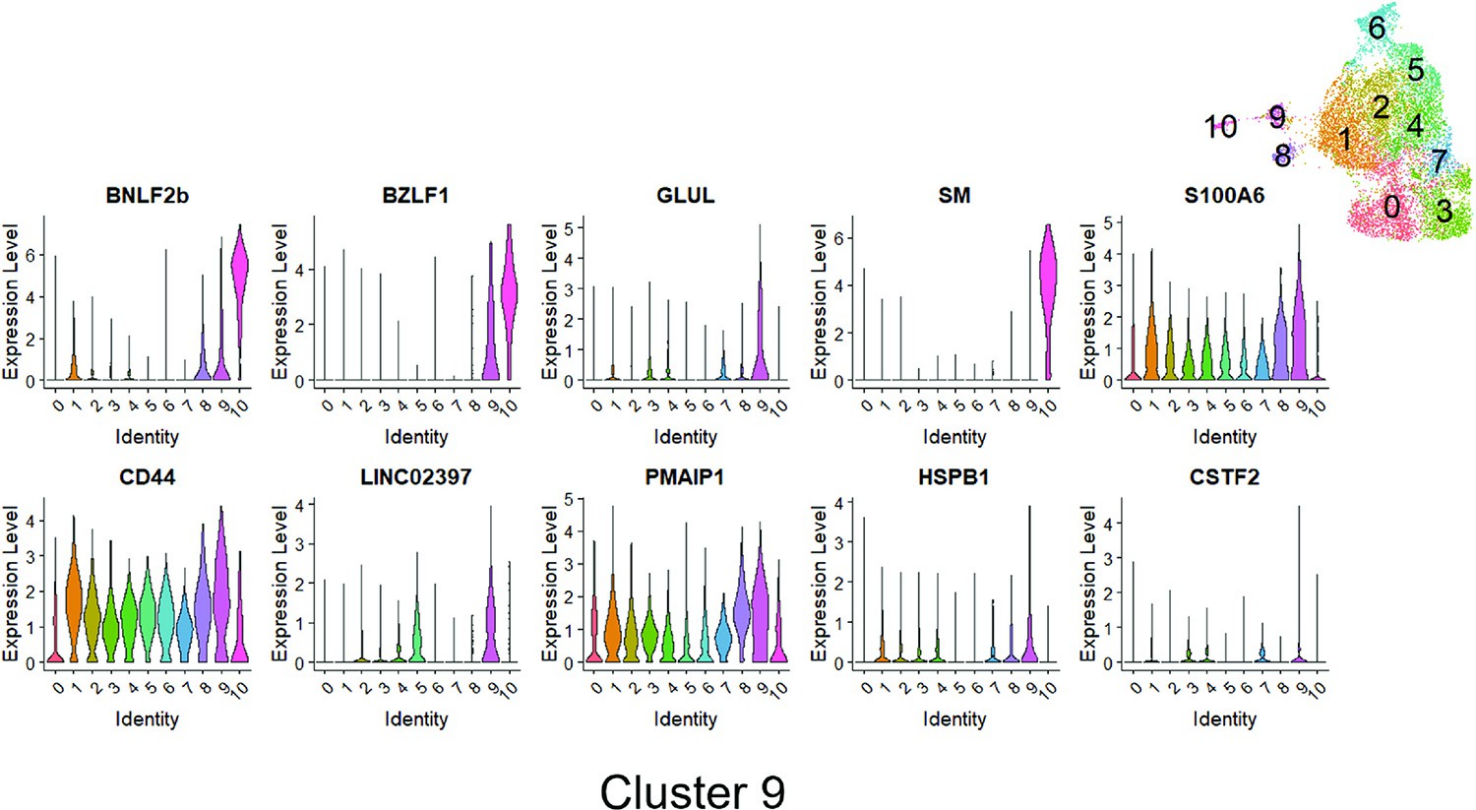
Gene expression analyses of Cluster 9. Violin plot of log normalized expression level of the top 10 differentially expressed genes sorted by average log2FC from MAST method (see Materials and Methods) across 11 identified clusters for Cluster 9 are shown.

**Fig 17:**
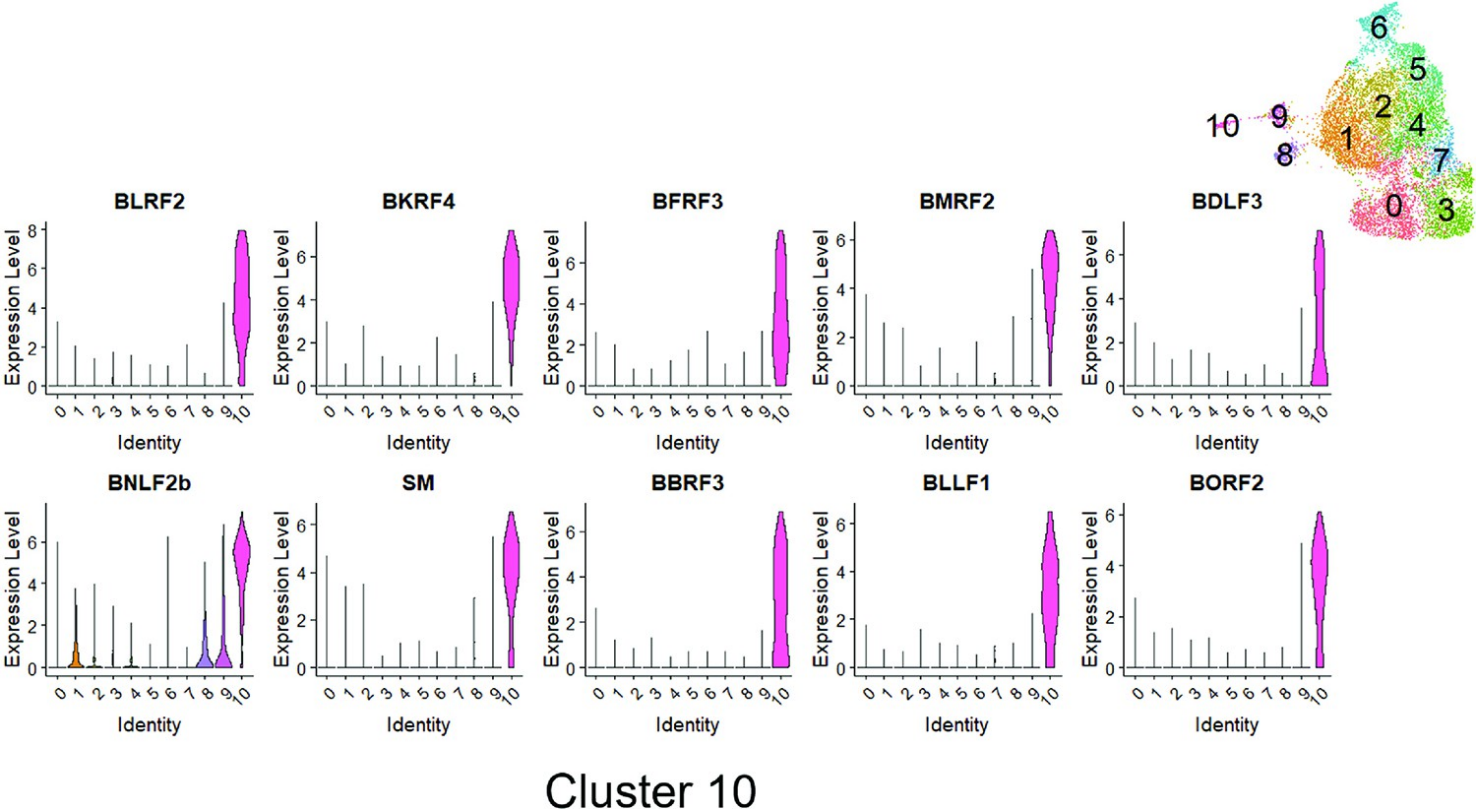
Gene expression analyses of Cluster 10. Violin plot of log normalized expression level of the top 10 differentially expressed genes sorted by average log2FC from MAST method (see Materials and Methods) across 11 identified clusters for Cluster 10 are shown.

**Fig 18:**
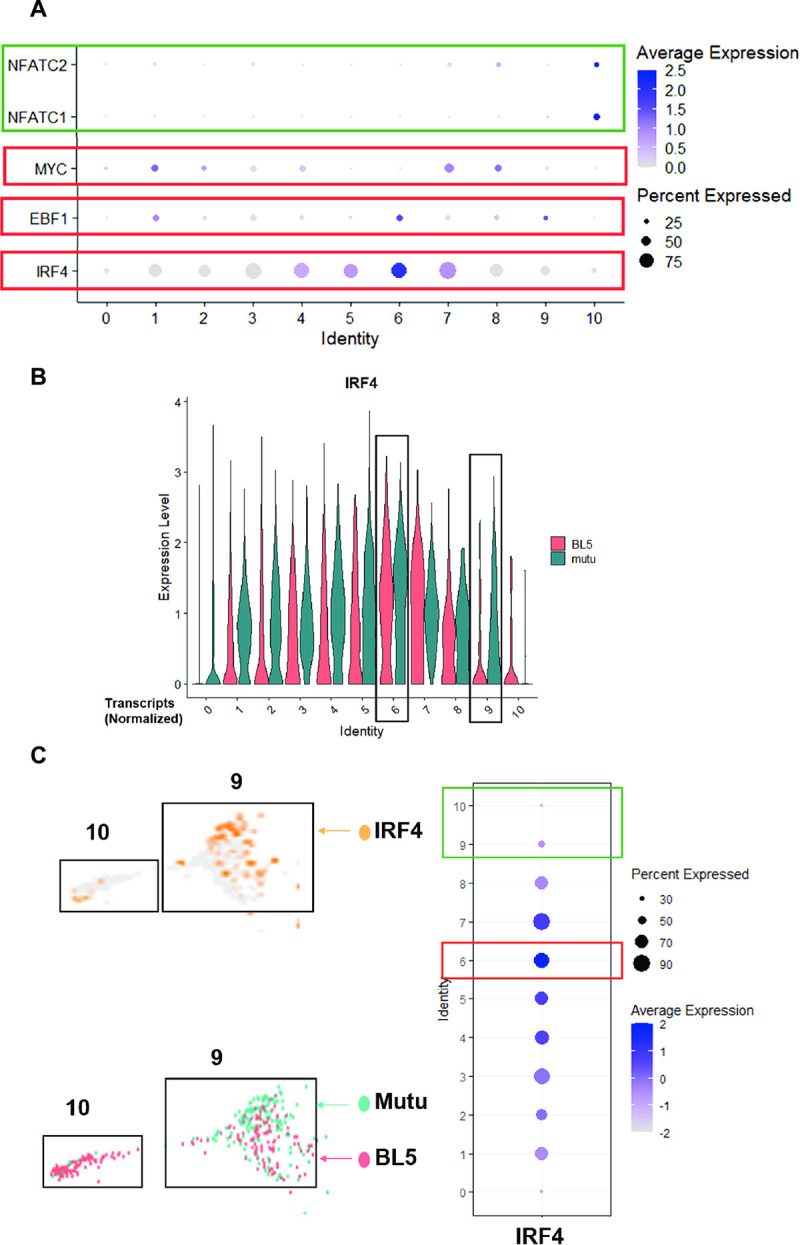
T2 LCLs have less IRF4 (Cluster 9) and more NFATc1 (Cluster 10) compared to latent cells, or T1 cells in Cluster 9. A. Dot plot presentation of NFATc1/c2 expression across all 11 cell clusters. **B.** Violin plot presentation of IRF4 expression in all 11 clusters shown separately for the BL5 and Mutu samples. **C.** Overlaid UMAP for expression of IRF4 in Cluster 9 and 10 split by sample type and DotPlot of IRF4 across all 11 clusters.

Lytically infected cells were distributed in two clusters, 9 and 10. Cluster 9 represents cells that have just started to enter lytic EBV infection, since they express some BZLF1 transcript (although much less than that observed in fully lytic cluster 10), but do not yet express most other lytic transcripts (**Figs 16 and 17**). Somewhat surprisingly, given the previously demonstrated ability of both BLIMP1 and XBP1 to activate the BZLF1 promoter in reporter gene assays and to induce lytic reactivation in some EBV-infected cell lines [7,8,10], we did not find lytic EBV transcripts in the plasmablast-like cluster 6 of T2 LCLs (**Figs 13 and 14)**. This unexpected finding may reflect the high level of IRF4 expression (which induces BLIMP1 expression) in cluster 6 (**Fig 18A**).

Two of the most over-represented cellular genes in Cluster 9 (CD44 and PMAIP) are known to be activated by BCR stimulation [50,51] (**Fig 16**), suggesting that this cluster may represent cells that have particularly high BCR activity. Surprisingly, T1 and T2 LCLs were similarly represented in Cluster 9 (**Fig 12**), suggesting that T1 LCLs in Cluster 9 are less able to proceed to fully lytic cluster 10 even if they begin to express BZLF1. To determine if this difference could be due to increased IRF4 expression in T1 versus T2 EBV-infected cluster 9 cells, we compared the levels of IRF4 expression in T1 versus T2 Cluster 9 cells (**Fig 18B).** Importantly, T1 cells in cluster 9 clearly expressed more IRF4 than T2 LCLs in cluster 9, strongly suggesting that persistent IRF4 expression in T1 cluster 9 cells contributes to their decreased ability to enter fully lytic infection. The geographic differences in IRF4 expression in T1 versus T2 Cluster 9 cells is shown in **Fig 18C**.

Of note, in addition to decreased expression of IRF4 in Cluster 10, both NFATc1 and NFATc2 expression were increased in the fully lytic Cluster 10 (**Fig 18A**). EBF1 expression was also decreased in the fully lytic Cluster 10 (**Fig 18A**). Overall, scRNA-seq data confirm that T2 LCLs are much more likely to enter fully lytic infection than T1 LCLs, and suggest that both viral and cellular factors (including NFATc1, IRF4 and EBF1 levels) contribute to this difference.

## Discussion

There are two major types of EBV, T1 and T2, but much less is known about T2 EBV compared to T1 EBV. Given that the major differences in the T1 versus T2 EBV genome are concentrated within the viral latency proteins EBNA2, EBNA3A, EBNA3B and EBNA3C, and that three of these proteins (EBNA2, EBNA3A and EBNA3C) are required for efficient T1 EBV transformation of B cells *in vitro*, the interaction of T2 EBV with the host B cell may be fundamentally different in comparison to T1 EBV. Although T2 EBV is known to transform B cells less efficiently than T1 EBV *in vitro*, a phenotype which has been attributed primarily to differences in T1 versus T2 EBNA2 functions [18], to date no in-depth analysis has been performed using genome-wide techniques (such as bulk and single cell RNA-seq) to compare the effects of T1 versus T2 EBV infection on global cellular gene expression in B cells. In addition, the mechanism(s) leading to increased lytic infection of T2 EBV-infected B cells [24] have not yet been fully explored. In this study, we have compared the effects of T1 versus T2 EBV infection on cellular gene expression in early-passage lymphoblastoid cell lines (obtained from a single donor) using both bulk and single-cell RNA-seq analysis. We find that T1- versus T2- infected B cell lines have very distinct differences in cellular gene expression, suggesting that T1 and T2 viruses have adopted different strategies for interacting with the host B cell. In addition, our results reveal that the increased lytic infection in T2 EBV-infected B cells is due to a variety of different viral and cellular differences between T1 EBV- and T2 EBV-infected B cells, including increased NFATc1 activity, and decreased IRF4 and EBF1 expression, in T2 LCLs, as well as the universal presence of the NFATc1-responsive Zp-V3 BZLF1 IE promoter variant in the T2 viral genome.

We found that four different independently generated LCLs infected with either the T1 Mutu strain or T1 Akata strain viruses have remarkably similar gene expression patterns, with no cellular genes having significantly different expression levels in the Mutu virus versus Akata virus infected cells. Likewise, the four T2 LCLs generated with the T2 AG876 strain virus and T2 BL5 strain viruses are also remarkably similar in terms of cellular gene expression. The similar gene expression patterns in each of the four T1 LCLs, and each of the four T2 LCLs, likely reflects the fact that only early-passage LCLs were used in these studies, and that all lines were derived from a single donor.

However, the four T1 LCLs are obviously different from the four T2 LCLs from bulk RNA-seq analysis, with close to 600 cellular genes being differentially regulated (**S1 Table)**. Furthermore, GSEA indicates that in comparison to T1 LCLs, T2 LCLs have a signature of increased BCR activation and increased NFAT activity. Consistent with this, we found that the CD11C (ITGAX) gene, which is known to be induced by BCR stimulation [30], is upregulated in T2 LCLs (**Fig 6**), and that this effect is at least partially due to enhanced NFAT activity in T2 LCLs since it was reversed by two different NFAT inhibitor drugs.

By having both bulk and scRNA-seq in our studies, we also asked whether the differences in cellular gene expression between T1 versus T2 LCLs is primarily due to cellular gene expression changes occurring only in the lytically infected cells of T2 LCLs, or also reflects differences in gene expression patterns of the latently infected cells. For example, by examining the expression of ITGAX (CD11C) in T1 versus T2 cells in each of the 11 different single-cell RNA-seq clusters, we determined that ITGAX is primarily upregulated in latently infected T2 LCL clusters (**S6 Fig**). Using this approach, we found that almost all other differentially expressed cellular genes initially identified by bulk RNA-seq analysis in T1 versus T2 LCLs reflect changes in the levels of cellular gene expression in the latently infected cell clusters of T1 and T2 LCLs. This result is not unexpected, since even in T2 LCLs the lytically-infected cells in Cluster 10 represent fewer than 10 percent of the total cell population, and the host shut-off functions of fully lytic herpesvirus infection are expected to non-specifically turn off many cellular genes in lytic cells. The finding that BCR-induced genes such as ITGAX and TNFRSF9 are enhanced in the latently infected T2 B cells suggests that the BCR-stimulated phenotype occurs in the latent as well as lytic cell populations, and that even in T2 LCLs only a relatively small portion of BCR-stimulated cells proceed to the fully lytic state of infection.

Another major finding in our studies is that the cellular transcription factor, IRF4, is expressed at lower levels in T2 LCLs compared to T1 LCLs, and that IRF4 negatively regulates lytic EBV reactivation. The difference in IRF4 expression between T1 and T2 LCLs were confirmed at both the RNA and protein levels **(S1 Table** and **Fig 7A**) but was more prominent at the protein level. IRF4 plays multiple different roles in B cell biology in a context- and concentration- dependent manner [52,53]. High levels of IRF4 expression, in which IRF4 preferentially binds promoters as a homodimer, induce plasma cell differentiation by turning on the master regulator of plasma cell differentiation, PRDM1 (BLIMP1), and turning off the master regulator of germinal center identity, BCL6 [52,53]. In contrast, at lower levels of expression, in which IRF4 preferentially forms heterodimers with other transcription factors such as PU.1 and BATF, IRF4 promotes germinal center (GC) formation by increasing expression of the activation-induced cytidine deaminase (AICDA) gene [52,53].

IRF4 is an essential survival factor for T1 EBV-transformed LCLs [39,40], as well as many activated diffuse large B cell lymphomas (DLBCLs) [54,55]. Of note, T1 EBV uses multiple different mechanisms to activate IRF4 in LCLs, including EBNA2-mediated transcriptional activation [56], LMP1-induced IRF4 phosphorylation by the SRC kinase [57,58] and EBNA3C-mediated IRF4 protein stabilization [59]. Conversely, the T1 EBV EBNA3C viral transcription factor must interact directly with IRF4 to activate certain cellular gene promoters [60]. Furthermore, since genes that are activated more efficiently by type 1 EBNA2 versus type 2 EBNA2 (including LMP1) have been previously shown to be enriched for EICE motifs [19,20], the higher level of IRF4 in type 1 LCLs might also contribute to enhanced activation of these EBNA2-responsive genes in type 1 LCLs. Given the ability of IRF4 to activate plasma cell differentiation (which can result in loss of proliferation and death of the host cell), activated DLBCLs that require IRF4 for survival often contain inactivating mutations of the PRDM1 gene to prevent such differentiation [54,55]. Likewise, in EBV-transformed LCLs, and transgenic mouse models, the T1 EBV latency proteins, EBNA3A and EBNA3C, have been shown to inhibit PRDM1 transcription [61,62]. EBV-induced IRF2 in T1 LCLs has also been proposed to inhibit PRDM1 expression [39]. Whether any of the IRF4- and/or PRDMI- regulating functions of the T1 EBV proteins described above are altered in the T2 EBV versions of these proteins is as yet unknown but is certainly an important question.

In contrast to its essential survival function in activated DLBCLs, IRF4 also serves as an important tumor suppressor that inhibits the development of another type of B-cell malignancy, chronic lymphocytic leukemia (CLL) [42–45]. BCR signaling plays a key role in promoting the survival and growth of CLL tumors. Over-expression of IRF4 in CLL cells reduces BCR signaling, including BCR-induced AKT and ERK phosphorylation and calcium release, and this effect has been proposed to be mediated at least partially through decreased expression of SYK and IKAROS [41]. Furthermore, germline SNPs in the human IRF4 gene 3’ untranslated region that decrease its expression increase susceptibility to CLL [63], and reduction of IRF4 expression promotes CLL in various mouse models [42–45]. We show here that IRF4 knock-down in T1 LCLs infected with Akata EBV promotes lytic EBV reactivation (**Fig 9A)**, while over-expression of IRF4 in the Akata Burkitt lymphoma cell line (which do not normally express IRF4) inhibits lytic EBV reactivation (**Fig 9B**). Furthermore, we find that co-transfection of an IRF4 expression vector inhibits both constitutive, and LMP2A-induced, ZpV3 promoter activity in EBV-negative Burkitt cells (**Fig 9C**). Consistent with our previous report that LMP2A-mediated activation of the ZpV3 promoter (like BCR-induced activation) is mediated by NFAT proteins [26], we also found that IRF4 expression in EBV-positive and EBV-negative Burkitt cell lines inhibits expression of both the NFATc1 and NFATc2 proteins (**Fig 10A** and **10B**). Thus, in addition to its ability to inhibit calcium release in response to BCR signaling (required for NFAT activity) our results here suggest that IRF4 also inhibits BCR signaling (and lytic EBV reactivation) by decreasing expression of the NFATc1 and NFATc2 proteins. In contrast to our results here, another group previously reported that IRF4 promotes lytic EBV reactivation in Burkitt cells [64]. Although we are uncertain why the previous report obtained different results, we suspect that non-physiologic levels of over-expressed IRF4 in the previous paper may have contributed, since high levels of IRF4 primarily promote IRF4 binding to promoters via the ISRE motif, while lower levels primarily promote IRF4 binding as heterodimers with BATF or PU.1 to IRF4-Ets1 (EICE) or IRF4-AP1 (AICE) composite motifs [52,53].

Importantly, GSEA of the gene expression patterns of T2 (versus T1) LCLs, and previously published gene expression patterns in IRF4-knockout (versus control) Type 1 LCLs [39] reveal substantial similarities in the upregulated genes (**Figs 8 and** **S3**). Most intriguingly, the previous published analysis showed that knockout of the IRF4 gene in Type 1 LCLs significantly increases NFATc1 and NFATc2 expression, as well as expression of the BCR-responsive ITGAX (CD11C) and TNFRSF9 genes **(S3 Fig and S4 Table**). Thus, we propose that increased IRF4 expression in T1 LCLs reduces lytic infection not only by attenuating BCR signaling, including NFAT activation, but also by decreasing NFATc1 and NFATc2 expression. Since the latent viral protein, LMP2A, mimics certain aspects of BCR signaling, including NFAT activation, while inhibiting other components of BCR signaling [65], it is possible that IRF4 also modulates some effects of LMP2A signaling which have downstream effects on lytic viral reactivation. Indeed, our finding that IRF4 inhibits LMP2A-mediated activation of a ZpV3-promoter construct (**Fig 9C**) suggests this is the case. Although the effects of LMP2A on lytic EBV reactivation are complex and context-dependent, with LMP2A appearing to induce lytic EBV reactivation in some contexts and inhibiting it in others, we previously showed that LMP2A protein levels are similar in T1 versus T2 LCLs [24] and thus differences in LMP2A expression do not appear to be responsible for the increased lytic phenotype of T2 LCLs. Of note, we were unable to obtain long-term IRF4 knockdown T2 LCLs, suggesting that similar to type 1 LCLs, IRF4 also functions as an essential survival factor in T2 LCLs, although it is expressed at lower levels.

An advantage of combining single-cell RNA-seq results with bulk RNA seq results is that the levels of IRF4 in T1 versus T2 LCLs within each of the 11 different single cell clusters can also be compared (**Fig 18**). This analysis revealed that the plasmablast-like cells in Cluster 6 have the highest level of IRF4 expression, and that the level of IRF4 is similar in T1 and T2 cells within this cluster. Given the known dependency of PRDM1 (BLIMP1) expression and plasma cell differentiation on IRF4 activity, it is perhaps not surprising that cluster 6 expresses much more IRF4 compared to the other clusters. Importantly, however, the high level of IRF4 in cluster 6 may help to explain why cells in this cluster remain latent even though they express high levels of two other transcription factors, PRDM1 and XBP1, that have been shown to activate the BZLF1 promoter and induce lytic EBV infection in other contexts.

Another important finding is that the T1 cells in cluster 9 (the cluster in which IE gene BZLF1 expression first occurs but the virus has not yet entered fully lytic infection) express much less IRF4 in comparison to the T2 cells (**Fig 18B**). Reduced IRF4 expression in T2 cluster 9 cells may explain why these cells are much more likely to progress to fully lytic infection (cluster 10) in comparison to T1 cluster 9 cells.

In addition to IRF4, another interesting transcription factor that we found to be downregulated in T2 LCLs in comparison to T1 LCLs is EBF1. The EBF1 transcription factor is a key regulator of many B-cell specific genes and is required for B-cell identity [66]. Like IRF4, EBF1 is essential for survival of T1 LCLs and its expression is activated by the latent viral protein EBNA1 [48]. In addition, a direct interaction between EBNA2 and EBF1 is required for the ability of EBNA2 to activate expression of some target cellular promoters [67,68]. Previously reported literature has already suggested that EBF1 may be a negative regulator of lytic EBV reactivation. For example, a survey of multiple different LCL lines found that high level expression of EBF1 is inversely correlated with lytic viral infection, and this inverse correlation is greater for EBF1 than for any other cellular gene [46]. More recently, EBF1 was reported to negatively regulate the expression of the BRLF1 IE gene promoter in reporter gene assays, and this effect was found to be strain type dependent [47]. Here we have shown that EBF1 inhibits constitutive activity of both the BZLF1 and BRLF1 IE promoters in AGS gastric cells **(S5 Fig**). Of note, EBF1 was recently shown to be a tumor suppressor in gastric carcinoma and is frequently inactivated in these tumors (including the AGS gastric carcinoma cell line) [69]. Thus, the loss of EBF1 expression in AGS cells may help to explain why EBV infection in this cell line is particularly lytic [70].

Although close to 600 cellular genes are differentially expressed in T1 versus T2 LCLs, and most of these differences in gene expression occur in the latently infected cells, the majority of the 10 different LCL clusters identified by scRNA-seq contained similar numbers of T1 and T2 cells, indicating that in addition to their differences, there are also many similarities between T1 and T2 LCLs. The characteristics of the latently infected clusters in this study that contain similar numbers of T1 and T2 cells are in many respects comparable to LCL clusters described in a recent paper that analyzed the signature of a number of different T1 LCLs using single-cell RNA-seq [71](**S7–S16 Figs)**. For example, both studies identify a cluster (Cluster 1 in this study) (**S8 Fig**) that expresses a high level of NF-kappa B target cellular genes and is presumed [71] or shown (**Figs 11 and 13** in this study) to have high level LMP1 expression. Similarly, both studies identify a cluster (Cluster 6 in this study) that has plasmablast-like features and both studies find that the plasmablast-like cluster is not a source of lytic EBV reactivation. Of note, we show here that this plasmablast-like cluster is over-represented in T2 LCLs compared to T1 LCLs (**Fig 12**) and propose that the high level of IRF4 expressed in this cluster may prevent lytic viral reactivation even in the presence of PRDM1(BLIMP1) and XBP1 expression. Interestingly, we also show here that the plasmablast-like cluster expresses very little EBNA2 or LMP1 (**Fig 13**), suggesting that one or both proteins may down-regulate plasma cell differentiation. Another cluster identified by both studies is a cluster (Cluster 3 in this study) (**Figs 11 and** **S10)**, characterized by high level KI67 and histone gene expression, likely representing replicating cells.

Remarkably, both studies also identify particularly high-level expression of the same set of specific cellular genes in the lytically-infected cell clusters, including NFATc1, MIER2, SFN, NHLH1 and SGK1 (**S17 Fig**). While NFATc1 activity is primarily regulated through post-transcriptional mechanisms, the promoter driving transcription of the shortest isoform of NFATc1 is driven by BCR activation [72]. Thus, the increased level of NFATc1 transcript in the most lytic cell cluster in both studies is consistent with increased BCR activation in these clusters. While our previous results suggest that over-expression of NFATc1 in the lytically infected clusters contributes to viral reactivation [24], it is as yet unknown whether the other cellular genes that are over-represented in the lytic clusters in both studies contribute to lytic reactivation or are simply the by-product of it. Finally, both studies also identify clusters (Cluster 0 in this study) that express few EBV genes and few B-cell markers (**Figs 11** and **S18**). The exact function and biologic relevance of this cluster has yet to be established.

In summary, we present here a global analysis of differential cellular and viral gene expression in B cells infected with T1 versus T2 EBV and show that there are numerous consistent differences in the effects of T1 versus T2 EBV infection on B cell gene expression. Although we have identified differences in IRF4 and EBF1 expression as likely contributors to the enhanced lytic infection in T2 LCLs, the biologic effects of multiple other differentially expressed genes in T1 versus T2 EBV infected B cells remain to be studied. For example, since caspase 1 activity is reported to be required for lytic induction by inflammasome stimulating agents in T1 LCLs [73], and we show here that caspase 1 expression is much reduced in T2 LCLs (**Fig 6B**), it will be critical to determine in the future whether T2 LCLs are relatively resistant to the lytic inducing effects of inflammasome activation. In addition, since we found that T1 and T2 LCLs have numerous differences in the expression levels of genes that encode various different cytokines, chemokines and their receptors (**S2 and S3 Tables**), it will be important in future studies to dissect the functional effects of these differences. Finally, the roles of various Type 1 and Type 2 EBV latency proteins in mediating these differences in gene expression will need to be determined. Most importantly, how these differences in the gene expression patterns of infected B cells affect the functions and pathogenesis of the Type 1 versus Type 2 EBV viruses in infected humans needs to be further defined.

## Materials and methods

### Cell lines and production of infectious EBV

The T1 EBV Akata-Bx1 Burkitt lymphoma cell line (originally derived by the Takada lab [74]) was a gift from Lindsay Hutt-Fletcher and was obtained by super-infecting an EBV-negative clone of Akata Burkitt lymphoma cells with recombinant Akata EBV containing a G418 resistance gene cassette and GFP gene inserted into the EBV BXLF1 gene as previously described) [75]. Type 1 EBV infected Mutu-1 cells, originally derived by the Rickinson lab at the University of Birmingham, UK [76], is a Burkitt lymphoma cell line (obtained as a gift from Jeff Sample). The Type 2 EBV-infected Burkitt lymphoma cell line AG876, originally derived by Pizzo *et al* [77], was obtained as a gift from Dr. Bill Sugden at the University of Wisconsin Madison. The Type 2 EBV-infected BL5 Burkitt lymphoma cell line, originally derived by the Harrington lab, was obtained as a gift from Rosemary Rochford at the University of Colorado [25,78]. The EBV negative Burkitt lymphoma line BJAB was purchased from ATCC. The EBV-negative version of the Akata Burkitt lymphoma cell line was a gift from Lindsey Hutt-Fletcher. All Burkitt lymphoma cell lines were maintained in RPMI media (Gibco) containing 10% fetal bovine serum (FBS), and 1% penicillin-streptomycin (pen/strep); Akata-Bx1 cells also were treated with 500 μg per ml of G418.

AGS-Akata cells are AGS cells stably infected with the Akata strain of EBV (containing a G418 resistance gene cassette and GFP gene inserted into the EBV BXLF1 gene derived as previously described [75] and were a kind gift from Lindsay Hutt-Fletcher. AGS and AGS-Akata cells were grown in F-12 media (Gibco) with 10% FBS and 1% pen/strep. Both the uninfected and EBV-infected AGS were cured (in the Hutt-Fletcher lab) of the contaminating SV5 virus present in most AGS lines. HeLa (a human cervical carcinoma cell line) was obtained from the American Type Culture Collection (Rockville, MD) and grown in DMEM media (Gibco) with 10% FBS and 1% pen/strep. 293 FT cells (ATCC) were maintained in DMEM media (Gibco) with sodium pyruvate (Gibco), non-essential amino acids (Gibco), 10% FBS, 500 μg per ml of G418, and 1% pen/strep.

EBV positive LCL lines were obtained by transforming peripheral blood B from a single donor cells with T1 (Akata and Mutu) and T2 (AG876 and BL5) EBV strains as described below. T1 and T2 EBV LCLs were grown in RPMI media containing 10% FBS and 1% pen/strep.

To produce infectious virus, BL cells were either treated with either 20 ng/ml phorbol-12-myristate3-acetate (PMA) (Sigma) and 3mM sodium butyrate (Sigma), or stably infected with a retrovirus expressing the EBV BZLF1 protein fused to the hormone domain of the estrogen receptor (Z-HT), constructed as previously described [79] (a gift from Dr. Bill Sugden, University of Wisconsin-Madison) and treated with 200 nM of 4-HT (Sigma) for 72h. Cell supernatants were harvested after 4 days of stimulation with 4-HT or PMA and sodium butyrate, and concentrated using a Sorvall GSA rotor at 16,000 g for 90 min. Virus was reconstituted at 1:200 initial volume in RPMI.

### Generation of lymphoblastoid cell lines (LCLs)

All LCLs used in these studies were derived from the same donor and generally used within 3 months of infection. To generate LCLs, adult peripheral blood mononuclear cells were isolated by Ficoll gradient, infected with four different virus types (Akata, Mutu, AG876, BL5), and multiple lines derived from each virus type. All RNA-seq experiments used cells infected with each virus type on the same day.

### Bulk RNA-seq analysis of T1 and T2 LCLs

Bulk RNA-seq libraries were prepared as previously described [80]. Briefly, lymphoblastoid cell lines were harvested in TRIzol or TRIzol LS (Invitrogen). RNA was isolated using the Direct-zol RNA MiniPrep Kit (Zymo Research) and RNA quality was assessed using an Agilent TapeStation. Ribodepleted library preparation using the Swift Rapid library prep kit and sequencing on an Illumina NovaSeq 6000 with 50-bp paired-end reads was performed by the Oklahoma Medical Research Foundation Clinical Genomics Center (Oklahoma City, OK). RNA-seq analysis of host transcription was conducted by BioInfoRx (Madison, WI) as previously described [80]. Briefly, fastQC was used to verify raw data quality of the Illumina reads. Reads were aligned to the GRCh38 (hg38) human genome primary assembly using Subjunc aligner from Subread [81] and assigned to genes using Ensembl annotation (v93). Raw counts were normalized using the TMM normalization method [82] using edgeR and the normalized gene counts were transformed to log2 scale using the voom method from the R Limma package [83], then used for differential expression analysis. Functional interpretation of the differentially expressed genes was conducted based on GO terms, KEGG pathway and GSEA [84,85] methods. For IRF4 gene set enrichment analysis, a gene set comprised of genes upregulated in Type 1 LCLs expressing IRF4 sgRNAs compared to control sgRNAs from [39] was constructed using the criteria Log2FC > 1 and adjusted p value < 0.01. Genes from the bulk Type 2 versus Type 1 LCLs analysis performed as previously described were then ranked with the ranking metric: -log(p-value)*sign(Log2FC) and gene set enrichment analysis was performed with GSEA.

### Bulk EBV gene expression analysis

EBV transcripts were analyzed as previously described [80]. Briefly, bulk RNA-seq reads were aligned to a Type 1 EBV genome (NC_007605.1) or the AG876 Type 2 genome (DQ279927.1) using Burrows-Wheeler Aligner (BWA) (82). SAMtools was used to generate sorted BAM files. A pileup of aligned reads was constructed as a Wig file using Python. Data were visualized using the UCSC Genome Browser using track hubs for Type 1 EBV or AG876 and a bedfile annotation for the corresponding EBV genome.

### Heatmap generation

The heatmap in **Fig 1** was generated as follows. EBV gene expression levels were quantified in RPKM using the UTS method [86]. Hierarchical clustering was calculated and visualized using the Euclidean distance method with the ComplexHeatmap (v2.8.0) R package [87]. The heatmap in **S3 Fig** was produced with the ComplexHeatmap (v2.8.0) R package [87]. Row Z-score values for the TPM normalized data set consisting of the IRF4 knock-out and control T1 LCLs and the data set presented in this paper were calculated independently. Hierarchical clustering was then calculated and plotted with the Euclidean distance method.

### Single cell RNA-seq analysis

ScRNA-seq was performed at the University of Wisconsin Biotechnology Center using 10X Genomics 3’ Single Cell RNA-Seq. 9.38E+05 live Mutu cells and 9.15E+05 live BL5 cells were used for sequencing, with a depth of sequencing of 78,990 reads per cell. The filtered feature data, including RNA read count for Mutu and BL5 strain genes, from CellRanger output was used for single-cell analysis with Seurat v3 framework [88]. Cells with more than 20% of transcripts coming from mitochondrial genes were excluded from the analysis. Cells with fewer than 300 genes or 450 transcript counts or more than 120,000 transcript counts were also excluded from the analysis. We used DoubletFinder [89] to remove potential multiplets before the data integration of Type 1 and Type 2. Cell cycle scores were calculated using a curated list of genes and regressed during normalization for each file virus using SCTransform [90]. The transformed data was then used to integrate the two datasets with an anchor-based approach to account for potential technical variants. Principal components analysis (PCA) was applied to the integrated dataset with 20 first PCs being used for further analysis determined based on standard deviation saturation plot in Seurat package. Uniform manifold approximation and projection (UMAP, https://arxiv.org/abs/1802.03426) was used for visualizing cell in 2D embedded space and overlaid with clustering results and representative markers. Cell clustering was performed with a graph-based algorithm based on shared-nearest-neighbor graph implemented in function FindClusters in Seurat package. We evaluated and justified the robustness of clustering at different resolutions guided by hierarchical tree of clusters, representative markers and required at least 2% of total cells in each cluster as minimum. Differential expression analysis to identify gene signatures for each subset was done using the MAST [91], which uses generalized linear models for testing genes expressed between the final clusters. Visualization plot (UMAP, ViolinPlot, DotPlot) was done using Seurat package. All analysis was done in R and Bioconductor opensource framework.

### EBV genome analysis

EBV transcripts were analyzed as previously described [80]. Briefly, fastq files were aligned to either T1 EBV (NC_007605) or T2 EBV (NC_009334) reference using Burrows-Wheeler Aligner (BWA) [92]. SAMtools was used to generate a sorted SAM file and a pileup of aligned reads was constructed as a Wig file using Python.

### Immunoblots

Immunoblots were performed as previously described [93]. Briefly, cell lysates were harvested with sumo lysis buffer with protease inhibitors (cOmplete, Roche). Quantitation of protein concentration was conducted with a sumo protein assay (Biorad). The lysates were separated using a 10% polyacrylamide gel and then transferred onto a nitrocellulose membrane. The membranes were subsequently blocked with 5% milk consisting of .1% Tween 20 and 1X PBS for one hour. Membranes were then incubated with primary antibody overnight. The following day the antibodies were removed and the membrane was washed with wash buffer (1X PBS, 0.1% Tween 20) three times for 5 minutes. The membrane was then incubated with secondary antibody suspended in 5% milk for one hour, before washing with wash buffer three times for 10 minutes before treatment with ECL (Thermofisher) and imaging. Image Studio Lite software was used to quantify levels of proteins of interest relative to loading controls tubulin or actin in certain figures.

### Drug treatments

L-Kynurenine (L-Kyn) was purchased from Sigma (K8625), dissolved in DMSO, and used at 100μM. Cyclosporin A was purchased form Selleckchem (S2286), dissolved in DMSO, and used at 300nM. FK506 was purchased from Selleckchem (S5003) resuspended in DMSO, and used at 300nM. Anti-IgG (Sigma I5260) was used at 10μg/mL for 30 minutes to overnight. Control conditions were treated with equal amounts of DMSO.

### Antibodies

The following antibodies were used for immunoblot analyses in this study: anti-R rabbit polyclonal antibody directed against the R peptide (peptide sequence EDPDEETSSQAVKALREMAD), anti-BZLF1 (Santa Cruz #sc-53904), anti-BMRF1 (Millipore #MAB8186), anti-IRF4 (Santa Cruz #sc-56713), anti-EBF1 (Biotechne #AF5165), anti-CD11C (Cell Signaling #45581), anti-caspase 1 (Abclonal #A0964), anti-ENPP2 (Proteintech #14243-1-AP), anti-Runx1 (Cell Signaling #4336S), anti-RUNX3 (Cell Signaling #13089), anti-NFATc1 (Santa Cruz #sc-7294), anti-NFATc2 (Cell Signaling #4389), anti-FYN (Santa Cruz #sc-434), anti-TNFRSF9 (Cell Signaling #34594), anti-CD9 (Cell Signaling #13174), anti-HLA DR/MHC class II (Santa Cruz #sc—53319), anti-AHR (Biotechne #AF6185), anti-CYP1B (Proteintech #18505-1-AP), anti-phospho-ERK (Cell Signaling #9101), anti-Tubulin (Sigma #T5168), anti-actin (Sigma #A5441), anti-V5 (Santa Cruz #sc—58052), and anti LMP2A (Santa Cruz #sc-101314). The secondary antibodies used were Horseradish peroxide (HRP)- labeled goat anti-mouse antibody (Fisher Scientific# 31431, 1:5000), HRP- labeled donkey anti-goat antibody (Fisher Scientific # A16005, 1:5000), HRP- labeled goat anti-rabbit antibody (Fisher Scientific# PI32460, 1:10000), and HRP-labeled goat anti-rat light-chain specific antibody (Millipore# AP202P).

### Plasmids

All plasmid DNA was prepared using the Qiagen Maxi-prep kit according to the manufacturer’s instructions. The plasmid pSG5 was purchased from Stratagene. pSG5-R and pSG5-Z (kind gifts from Diane Hayward of John Hopkins University) contain the BZLF1 (Z) and BRLF1 (R) immediate-early genes driven by the SV40 promoter as previously described [94]. The IRF4 expression vector (plenti-CAG-IRF4-FLAG-IRES-GFP) used in transfection experiments was a gift from William Kaelin (Addgene plasmid # 107389) [95]. The LMP2A expression vector was a gift from Richard Longnecker at Northwestern University. The IRF4 lentivirus vector with blasticidin resistance (IRF4-pLenti6.3/V5-DEST) that was used to create stable cell lines in uninfected and EBV-infected Akata Burkitt lymphoma cells was purchased from DNASU (#V53306). psPAX2 (Addgene #12260), phCMV-GALV-MTR (Addgene #163612), and pLKO.1-Blast (Addgene # 26655) were used to package the lentiviruses. The pcDNA-EBF1 expression vector was a gift from Bo Zhao of Brigham and Women’s Hospital and Harvard Medical School. The V5-tagged lentivirus EBF1 vector was obtained from DNASU (HsCD00943381). The pCpG Zp-83 luciferase, pCpG Zp-668 luciferase, and pCpG Rp-673 vectors were all described previously [10,96].

### shRNAs

Plasmids containing shRNA targeting EBF1 (TRCN0000013828, TRCN0000013829, TRCN0000013830, TRCN0000013831, and TRCN0000013832) and IRF4 (TRCN0000014763, TRCN0000014764, TRCN0000014765, TRCN0000014766, and TRCN0000014767) on a pLKO backbone were obtained from Hozizon Discovery (Lafayette, CO, USA). Plasmids were grown and purified using Qiagen High-Speed Midi Kit. Four micrograms of purified lentivral plasmid were co-transfected with 1ug phCMV-GALV-MTR (Addgene #163612, Watertown, MA) and 3ug pSPAX2 (Addgene #12260) into 293FT (ThermoFisher) cells, grown in DMEM (Fisher Scientific, Pittsburgh, PA), 10%FBS (GeminiBio, Sacramento, CA), 1x non-essential amino acids DMEM (Fisher Scientific), 1x glutamine (Fisher Scientific), using 24ul of Lipofectamine 2000(Fisher Scientific) overnight as per manufacturer’s instructions. The following morning, the media was changed to RPMI (Fisher Scientific), 10% FBS, 1X Penn-Strep (Fisher Scientific) and culture continued a further 24 hours. Media was then harvested and 0.8u filtered. Cells were spinoculated by addition of polybrene (Millipore-Sigma, St. Louis MO) to 10ug/ml and centrifuging for 90 minutes at 1500g, room temperature, followed by resuspension in RPMI culturing for 72h-96h and then addition of puromycin to 1ug/ml and culturing a further 5–9 days.

### Transient transfections

DNA was transfected into AGS cells using the Lipofectamine 2000 (Thermo Fisher #11668019) system according to the manufacturer’s protocol. Generally, 500ng of total DNA with 1.5 μL Lipofectamine 2000 was used per condition to transfect epithelial cells that were approximately 70% confluent in a 12 well plate. BJAB Burkitt lymphoma cells were nucleofected using the Amaxa Nucleofector 2b device (Lonza) and program M-013 (with Buffer V) with 1–2 ug DNA per condition.

### Creation of Akata Burkitt lymphoma cell lines stably expressing IRF4

pLKO.1-Blast or IRF4-pLenti6.3/V5-DEST were transfected into 293 FT cells along with the packaging plasmids psPAX2 and phCMV-GALV-MTR. Media from cells was recovered after 24 hours, filtered with 0.8μM filter, and virus used to infect EBV-negative (-) or EBV-infected Akata Burkitt lymphomas. Cells were spinoculated for 90 minutes at 1500g with virus and 1μg/mL polybrene (Millipore #TR-1003-G). Cells were re-susupended in media and left to grow for 3–4 days before addition of blasticidin at 5ug/mL. Experiments were performed after stably infected cell lines were obtained.

### Luciferase reporter assays

Luciferase reporter assays in AGS cells were conducted as previously described [96] using Lipofectamine 2000. BJAB cells were nucleofected using the Amaxa Nucleofector 2b device (Lonza) and program M-013 (with Buffer V) with 1–2 ug DNA per condition. 48 hours after transfection or nucleofection cells were washed once with PBS, suspended in 150–200 μl of 1X reporter lysis buffer (Promega) and then flash-frozen once. After pelleting by centrifugation and removal of the supernatant to a new tube, luciferase assays were performed according to the manufacturer’s instruction using a BD Monolight 3010 luminometer (BD Biosciences). All experiments were done in duplicate and repeated at least twice in separate experiments.

### Flow cytometry

Cells were stained with Alexa Fluor 647-labeled anti-CD11c clone 3.9 (catalog # 301620, BioLegend, San Diego, CA) or isotype control mouse IgG1 clone MOPC-21 (catalog # 400130, BioLegend) at 50 μg/ml. Fluorescence was measured on a LSR II flow cytometer (BD Biosciences, Franklin Lakes, NJ) and data analysis performed with FlowJo 9.8 software (BD Biosciences).

## Supporting information

S1 Fig T2 LCLs express higher levels of RUNX1, FYN, CD137, CD9, MHC class II, CYP1B, and AHR compared to T1 LCLs. Immunoblot analyses were performed to compare the protein expression levels of various different cellular proteins including **A)** RUNX1 and RUNX3, **B)** FYN, **C)** CD137 **D)** CD9, **E)** MHC Class II (HLA-DR), **F)** CYP1B and AHR in T1 versus T2 LCLs as indicated. Actin or tubulin was used as loading control.
(PDF)

S2 Fig AHR activator L-Kyn suppresses Z expression in type 2 LCLs. T2 EBV infected LCLs were treated with or without an AHR activating compound, L-Kyn, for 6 days and the amount of lytic EBV Z protein expressed, as well as the level of AHR target CYP1B, was then assessed by immunoblot. Actin was used as loading control.
(PDF)

S3 Fig A Heat map of the GSEA shown in Fig 8. Genes upregulated in IRF4 knock-out type 1 LCLs versus genes upregulated in T2 versus T1 LCLs (p < 0.05) are shown, with some genes of interest indicated on the right side of the figure. Expression levels for each data set were independently computed as row Z-score values prior to hierarchical clustering to account for batch effects. This is emphasized by a gray vertical line between the two data sets. The heatmap was split into two groups to show significantly upregulated genes in the T2 versus T1 LCLs (adjusted p-value < 0.05, log2FC > 1) which are labeled with gene names.
(PDF)

S4 Fig IRF4 knock-down induces lytic EBV reactivation in Type 1 Akata LCLs. Akata LCLs were infected with two different sets of lentiviruses expressing IRF4 targeted shRNAs or a control shRNA vector, selected with puromycin for 4 days, and then immunoblot analysis was performed to detect expression of IRF4, the lytic viral protein BMRF1, or actin as indicated. The numbers below each immunoblot quantify the results using Image Studio Lite software to normalize the levels of IRF4 and BMRF1 expression to actin expression. Results are presented as the ratio of IRF4 and BMRF1 expression relative to actin in shIRF4 cells relative to vector control (pLKO) cells. Vector control values are set as 1.
(PDF)

S5 Fig EBF1 inhibits EBV BZLF1 and BRLF1 IE promoter activity. EBV negative gastric carcinoma AGS cells were transfected with luciferase reporter vectors driven by the intact BZLF1 promoter (Zp-668), a minimal BZLF1 promoter construct (Zp-83) (left), or the intact BRLF1 promoter (Rp-673) (right) in the presence or absence of a co-transfected EBF1 expression vector. The amount of luciferase activity produced by each condition is shown.
(PDF)

S6 Fig ITGAX expression in T2 LCLs is confined to latently infected clusters. The levels of ITGAX transcript in scRNA-seq results in T1 versus T2 clusters is shown.
(PDF)

S7 Fig Violin plot of log normalized expression level of the top 10 differentially expressed genes sorted by average log2FC from MAST method (see Materials and Methods) across 11 identified clusters for Cluster 0. (PDF)

S8 Fig Violin plot of log normalized expression level of the top 10 differentially expressed genes sorted by average log2FC from MAST method (see Materials and Methods) across 11 identified clusters for Cluster 1. (PDF)

S9 Fig Violin plot of log normalized expression level of the top 10 differentially expressed genes sorted by average log2FC from MAST method (see Materials and Methods) across 11 identified clusters for Cluster 2. (PDF)

S10 Fig Violin plot of log normalized expression level of the top 10 differentially expressed genes sorted by average log2FC from MAST method (see Materials and Methods) across 11 identified clusters for Cluster 3. (PDF)

S11 Fig Violin plot of log normalized expression level of the top 10 differentially expressed genes sorted by average log2FC from MAST method (see Materials and Methods) across 11 identified clusters for Cluster 4. (PDF)

S12 Fig Violin plot of log normalized expression level of the top 10 differentially expressed genes sorted by average log2FC from MAST method (see Materials and Methods) across 11 identified clusters for Cluster 5. (PDF)

S13 Fig Violin plot of log normalized expression level of the top 10 differentially expressed genes sorted by average log2FC from MAST method (see Materials and Methods) across 11 identified clusters for Cluster 6. (PDF)

S14 Fig Violin plot of log normalized expression level of the top 10 differentially expressed genes sorted by average log2FC from MAST method (see Materials and Methods) across 11 identified clusters for Cluster 7. (PDF)

S15 Fig Violin plot of log normalized expression level of the top 10 differentially expressed genes sorted by average log2FC from MAST method (see Materials and Methods) across 11 identified clusters for Cluster 8. (PDF)

S16 Fig Violin plot of log normalized expression level of the top 10 differentially expressed cellular genes sorted by average log2FC from MAST method (see Materials and Methods) across 11 identified clusters for Cluster 10. (PDF)

S17 Fig The gene expression patterns of various cellular genes shown to be most up-regulated in the lytic cluster of a previous scRNA-seq study [71] are shown in the various different clusters. (PDF)

S18 Fig The gene expression patterns of various cellular genes important to B cell biology are shown. (PDF)

S1 Table Bulk RNAseq data of Type 1 and Type 2 LCLs. (CSV)

S2 Table Examples of Genes upregulated in T2 versus T1 lymphoblastoid cell lines. Selected genes of interest that are upregulated in T2 LCLs compared to T1 LCLs in the RNA-seq results are shown, along with the fold-increase in gene expression and the adjusted p value.
(DOCX)

S3 Table Examples of cellular genes down-regulated in T2 LCLs versus T1 LCLs. Selected genes of interest that are downregulated in T2 LCLs compared to T1 LCLs in the RNA-seq results are shown, along with the fold-increase in gene expression and the adjusted p value.
(DOCX)

S4 Table Examples of Genes upregulated in both IRF4-KO (versus control) T1 LCLs and T2 (versus T1) LCLs. Selected genes of interest that are upregulated in IRF4-KO T1 LCLs and T2 LCLs are shown, along with the fold-increase in gene expression and the adjusted p value.
(DOCX)
