# Supplementary figures and images for: Reduced IRF4 expression promotes lytic phenotype in Type 2 EBV-infected B cells
Source: PLoS Pathog. 2022 Apr 26;18(4):e1010453. doi: 10.1371/journal.ppat.1010453 (PMC9041801; doi:10.1371/journal.ppat.1010453)

Supplemental Figure 1

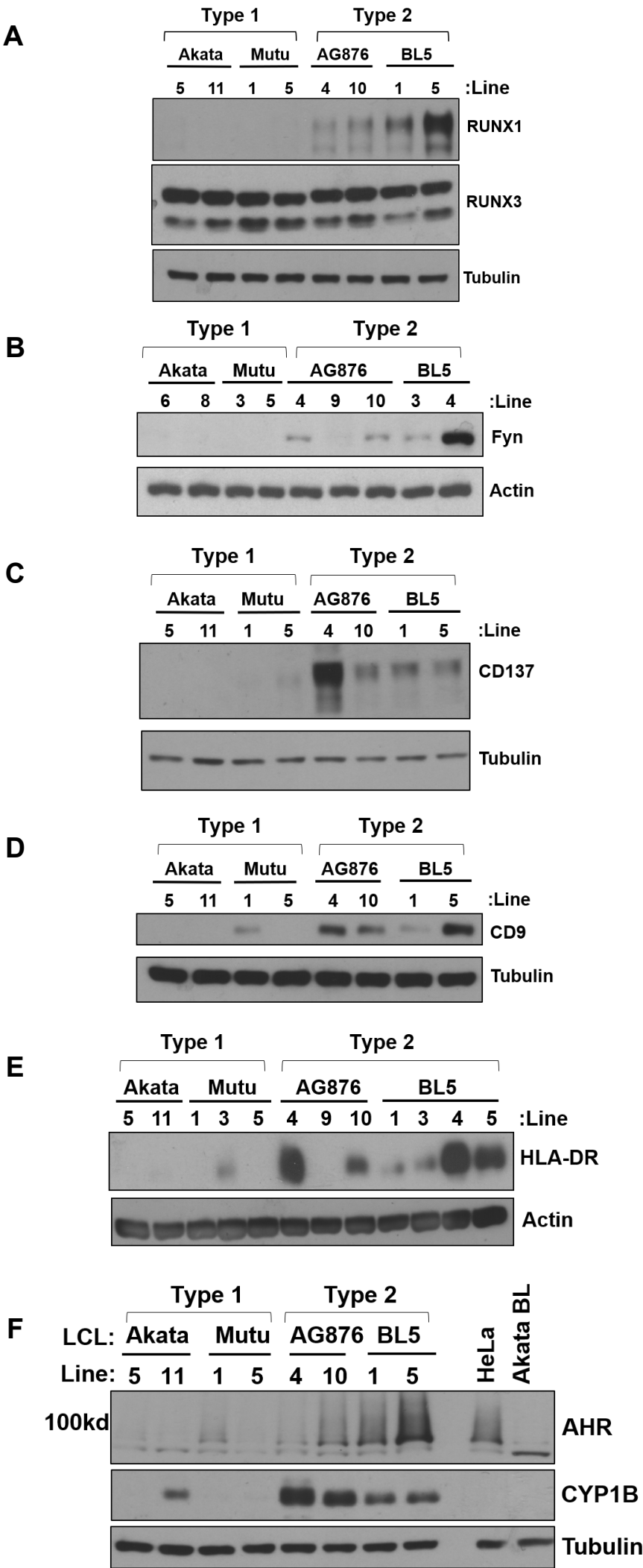

Supplement: S1 Fig — Immunoblot analyses were performed to compare the protein expression levels of various different cellular proteins including A) RUNX1 and RUNX3, B) FYN, C) CD137 D) CD9, E) MHC Class II (HLA-DR), F) CYP1B and AHR in T1 versus T2 LCLs as indicated. Actin or tubulin was used as loading control. (PDF) [file ppat.1010453.s001.pdf]

## Supplemental Figure 2

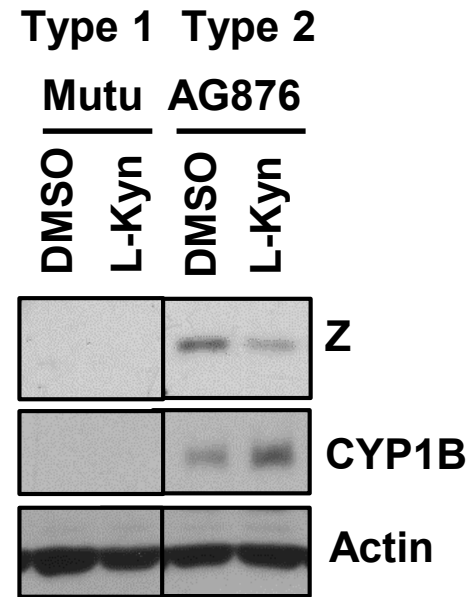

Supplement: S2 Fig — T2 EBV infected LCLs were treated with or without an AHR activating compound, L-Kyn, for 6 days and the amount of lytic EBV Z protein expressed, as well as the level of AHR target CYP1B, was then assessed by immunoblot. Actin was used as loading control. (PDF) [file ppat.1010453.s002.pdf]

Row Z-Score of  
TPM normalized values

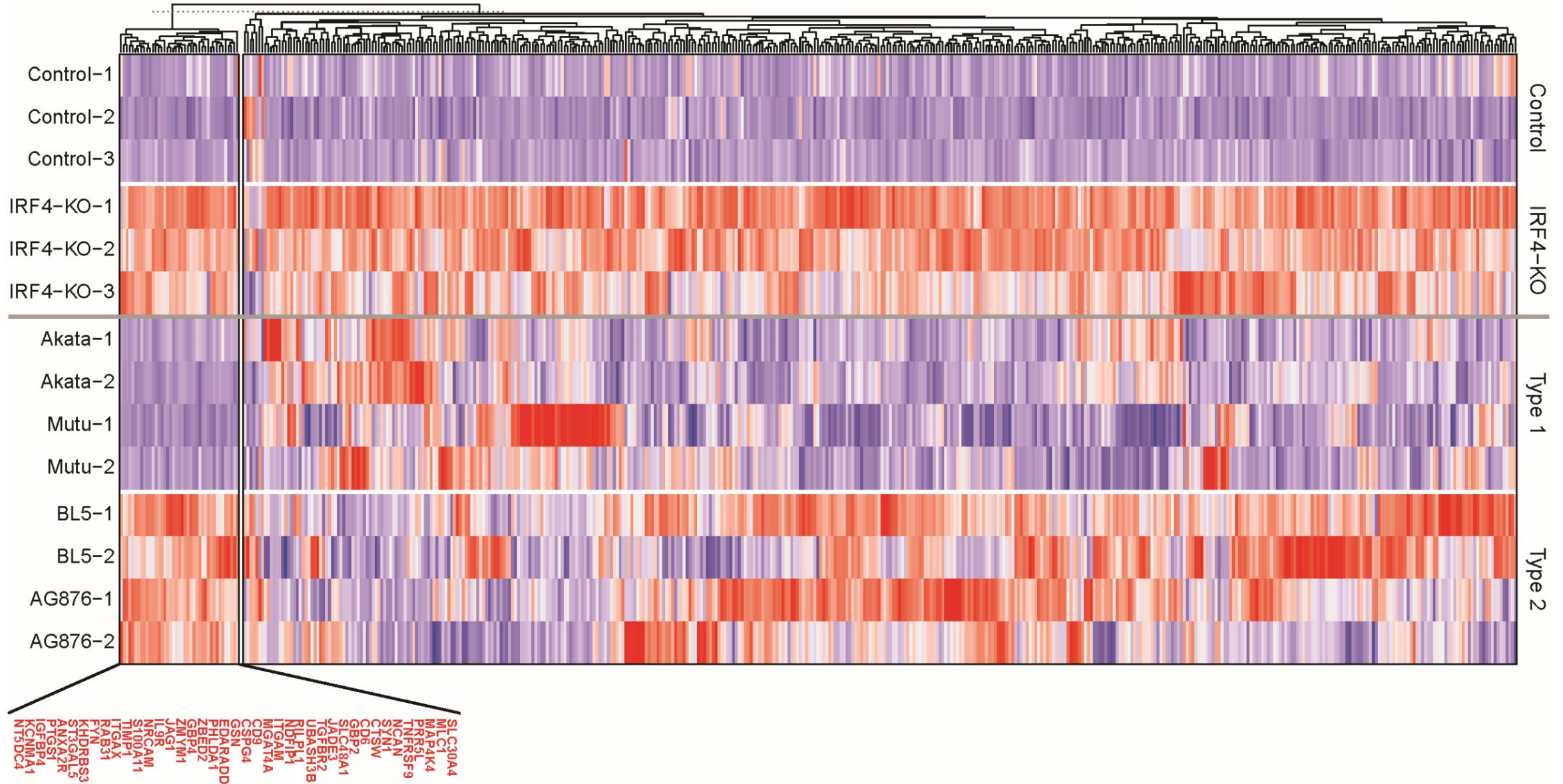

Supplement: S3 Fig — Genes upregulated in IRF4 knock-out type 1 LCLs versus genes upregulated in T2 versus T1 LCLs (p < 0.05) are shown, with some genes of interest indicated on the right side of the figure. Expression levels for each data set were independently computed as row Z-score values prior to hierarchical clustering to account for batch effects. This is emphasized by a gray vertical line between the two data sets. The heatmap was split into two groups to show significantly upregulated genes in the T2 versus T1 LCLs (adjusted p-value < 0.05, log2FC > 1) which are labeled with gene names. (PDF) [file ppat.1010453.s003.pdf]

# Supplemental Figure 4

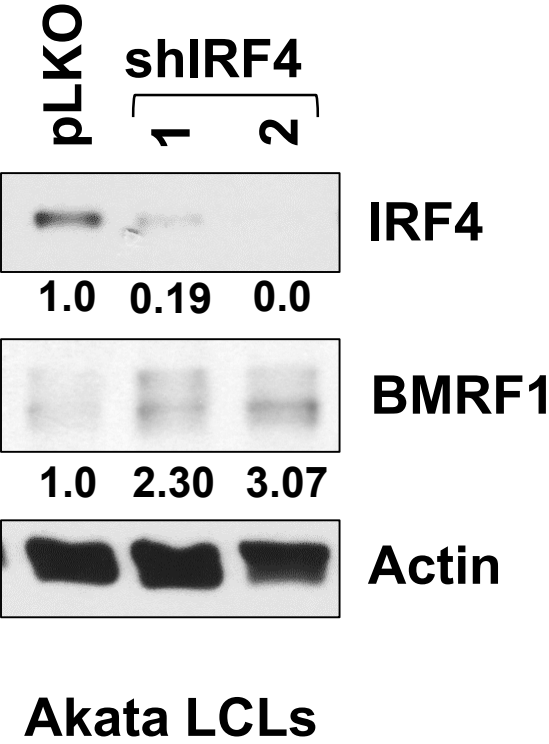

Supplement: S4 Fig — Akata LCLs were infected with two different sets of lentiviruses expressing IRF4 targeted shRNAs or a control shRNA vector, selected with puromycin for 4 days, and then immunoblot analysis was performed to detect expression of IRF4, the lytic viral protein BMRF1, or actin as indicated. The numbers below each immunoblot quantify the results using Image Studio Lite software to normalize the levels of IRF4 and BMRF1 expression to actin expression. Results are presented as the ratio of IRF4 and BMRF1 expression relative to actin in shIRF4 cells relative to vector control (pLKO) cells. Vector control values are set as 1. (PDF) [file ppat.1010453.s004.pdf]

Supplemental Figure 5

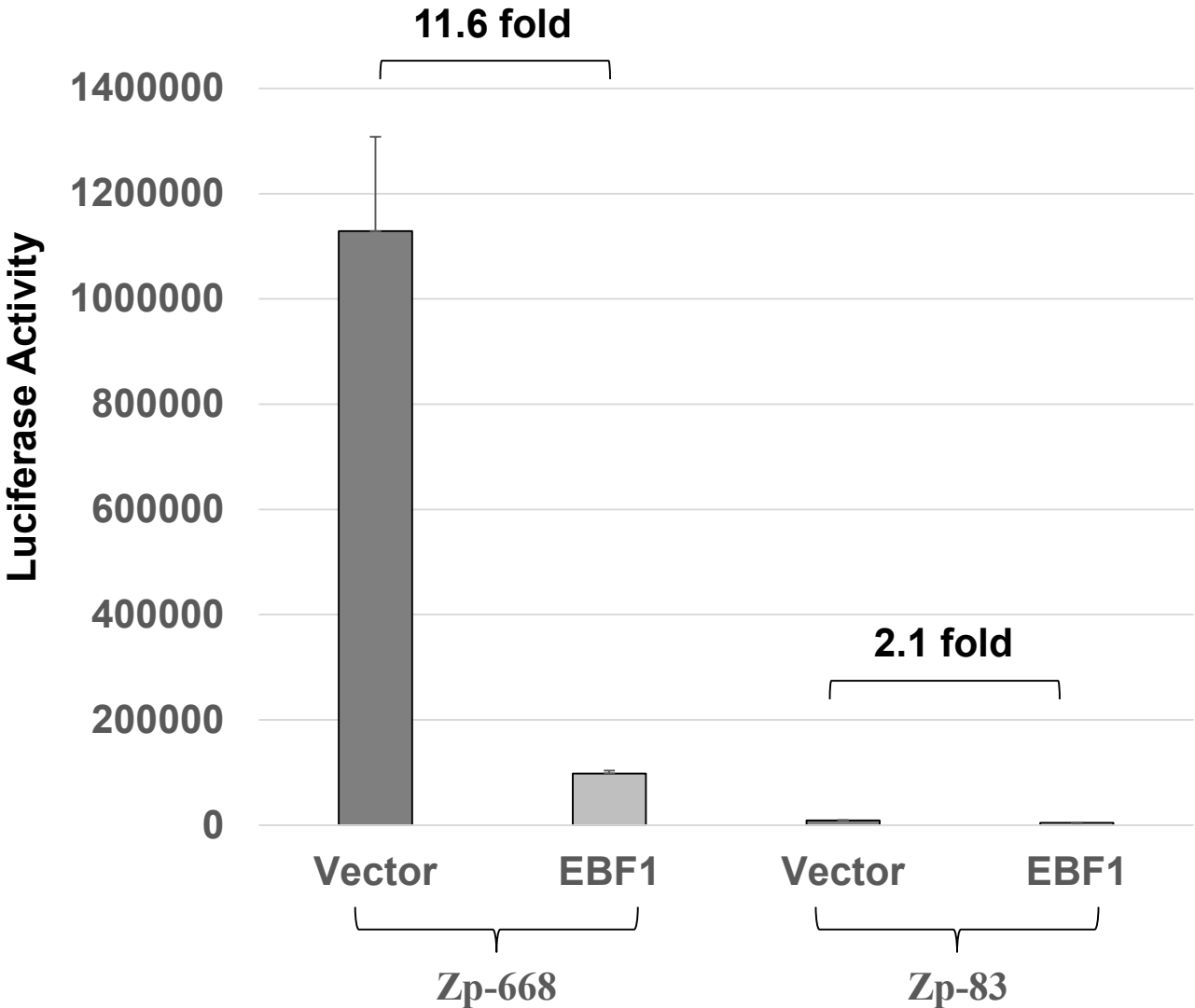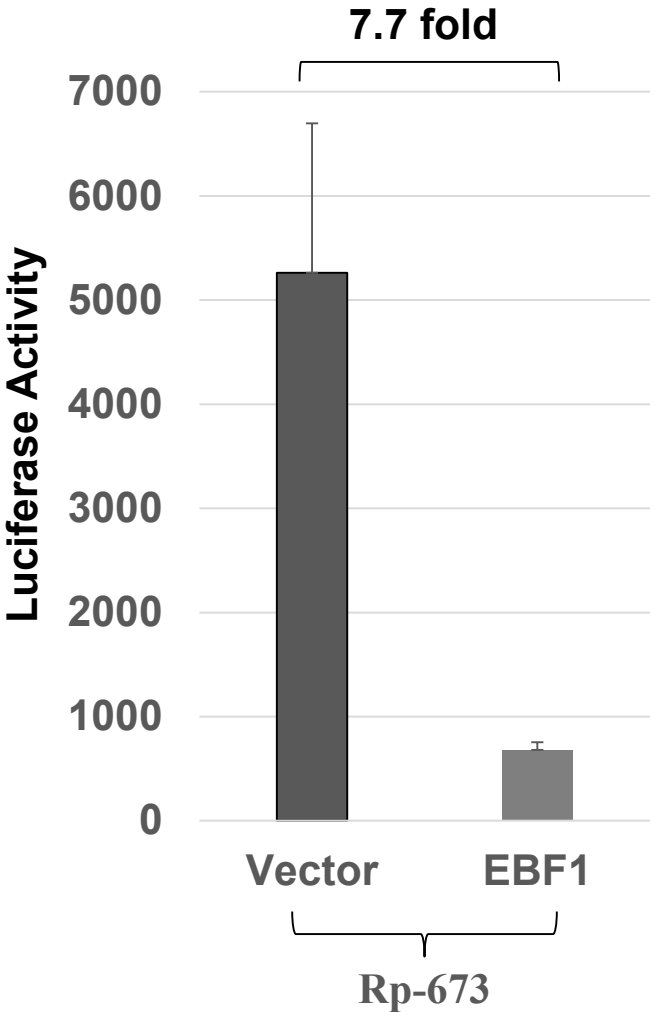

Supplement: S5 Fig — EBV negative gastric carcinoma AGS cells were transfected with luciferase reporter vectors driven by the intact BZLF1 promoter (Zp-668), a minimal BZLF1 promoter construct (Zp-83) (left), or the intact BRLF1 promoter (Rp-673) (right) in the presence or absence of a co-transfected EBF1 expression vector. The amount of luciferase activity produced by each condition is shown. (PDF) [file ppat.1010453.s005.pdf]

Supplemental Figure 6

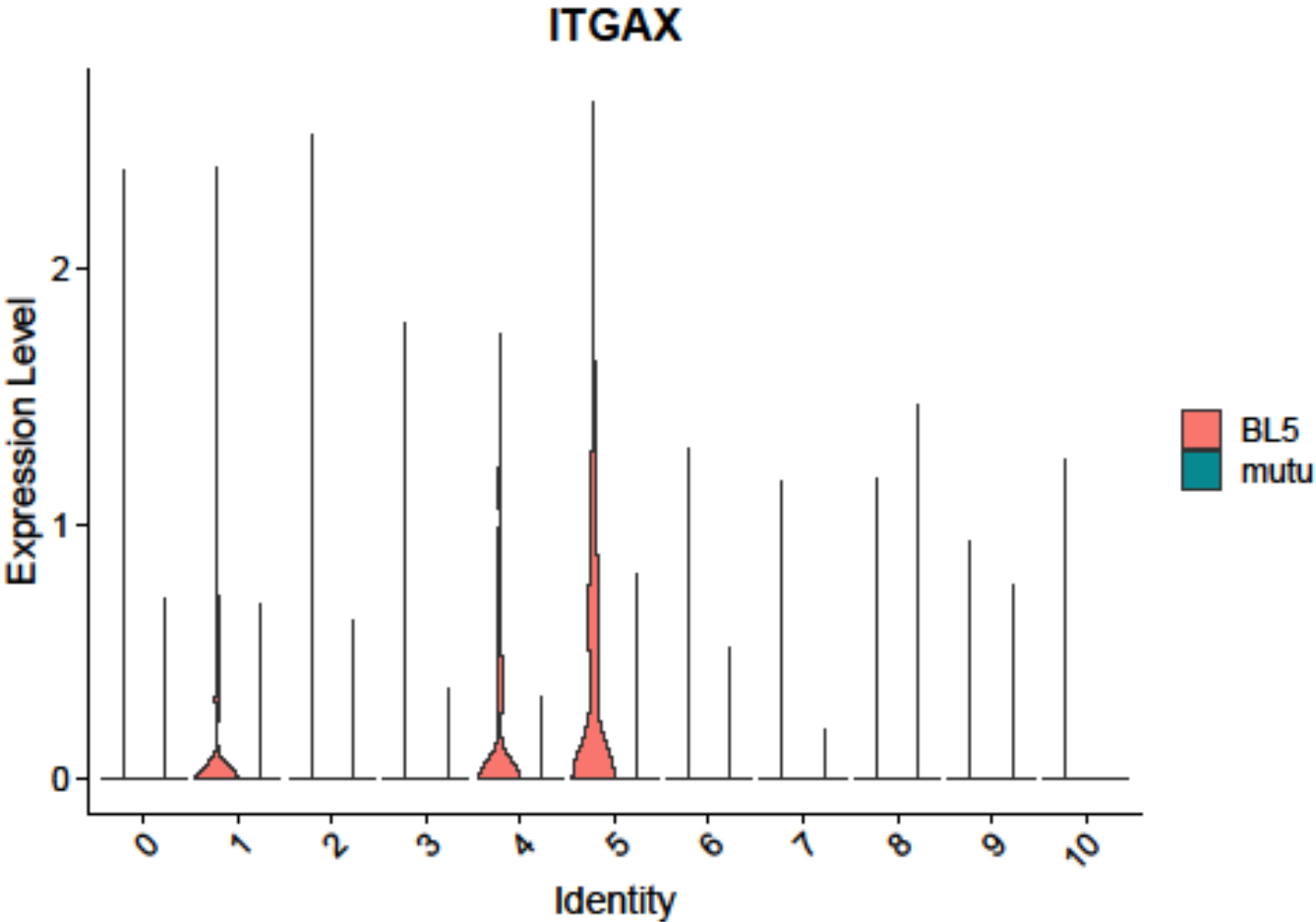

Supplement: S6 Fig — The levels of ITGAX transcript in scRNA-seq results in T1 versus T2 clusters is shown. (PDF) [file ppat.1010453.s006.pdf]

# Supplemental Figure 7

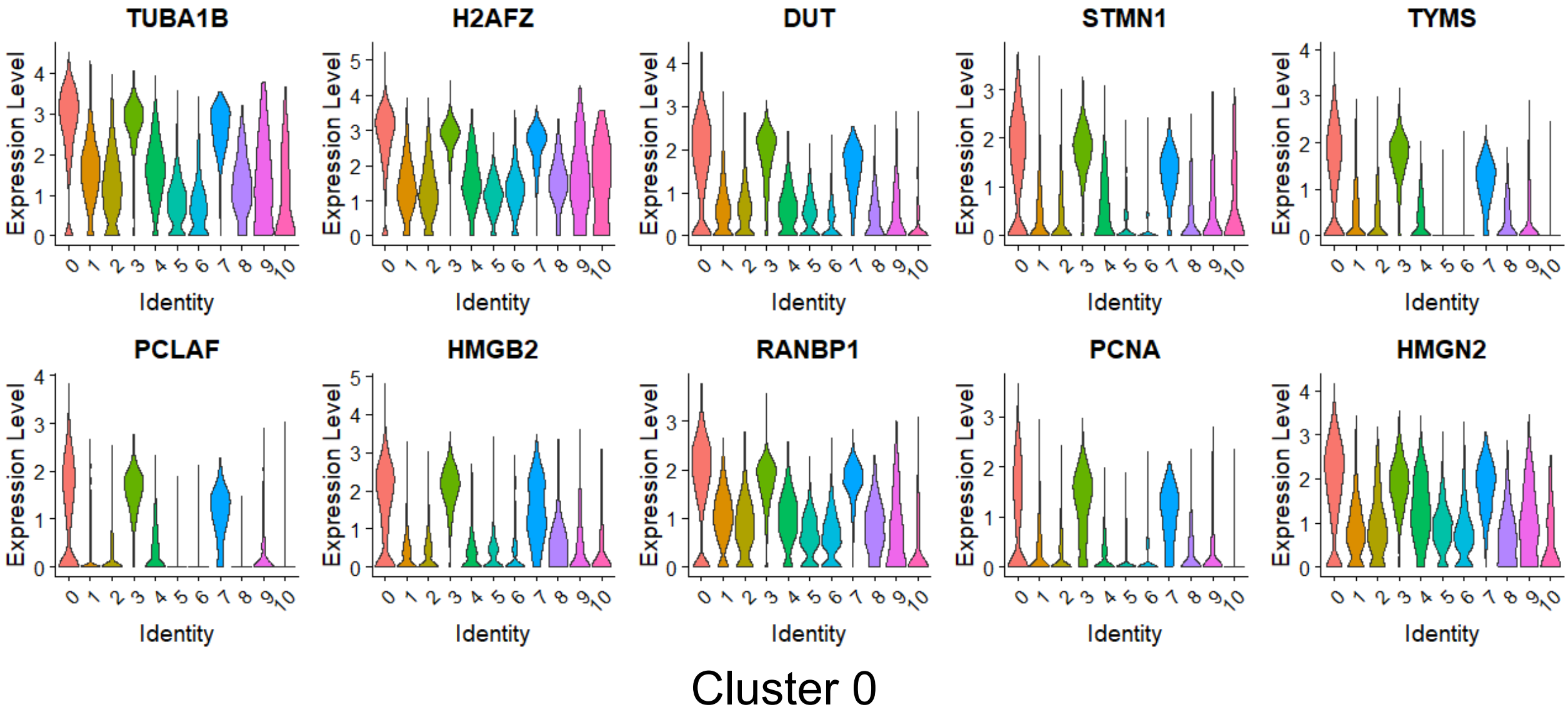

Supplement: S7 Fig — (PDF) [file ppat.1010453.s007.pdf]

# Supplemental Figure 8

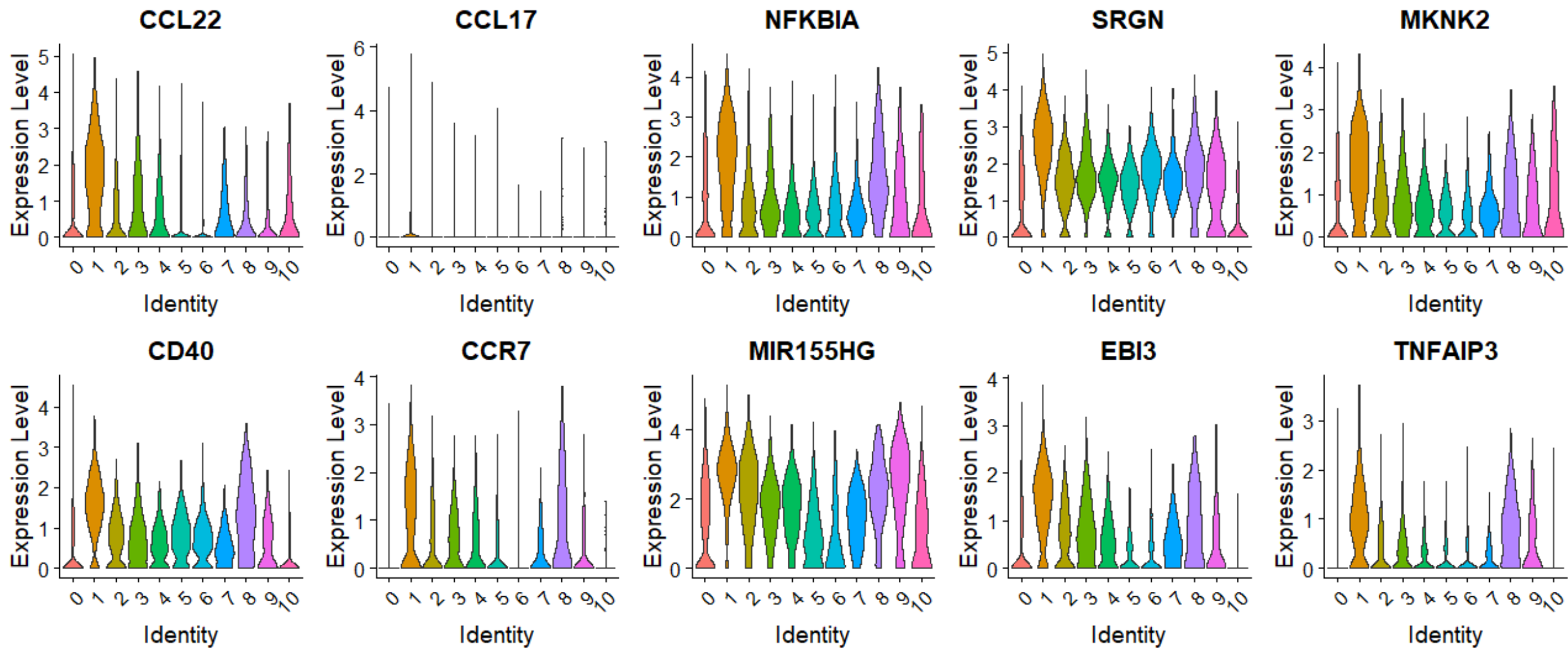

Cluster 1

Supplement: S8 Fig — (PDF) [file ppat.1010453.s008.pdf]

# Supplemental Figure 9

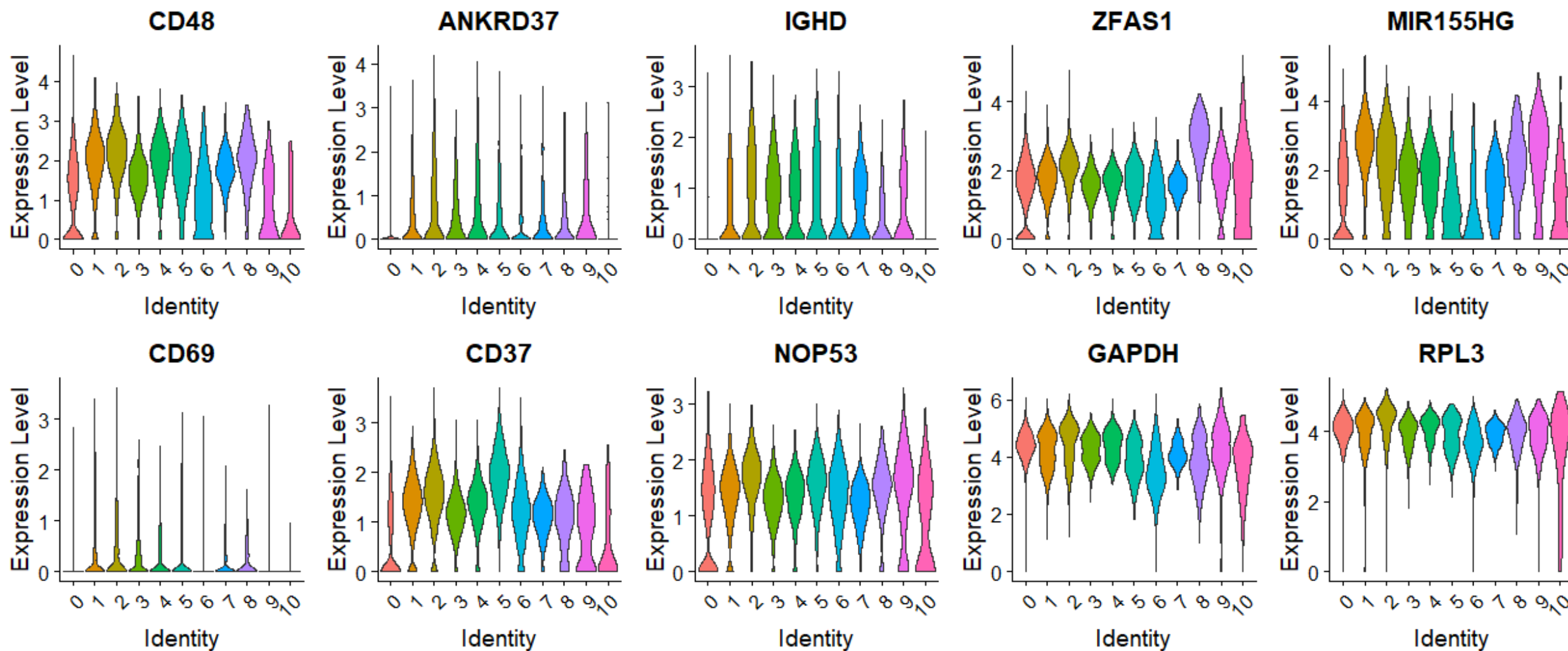

Cluster 2

Supplement: S9 Fig — (PDF) [file ppat.1010453.s009.pdf]

# Supplemental Figure 10

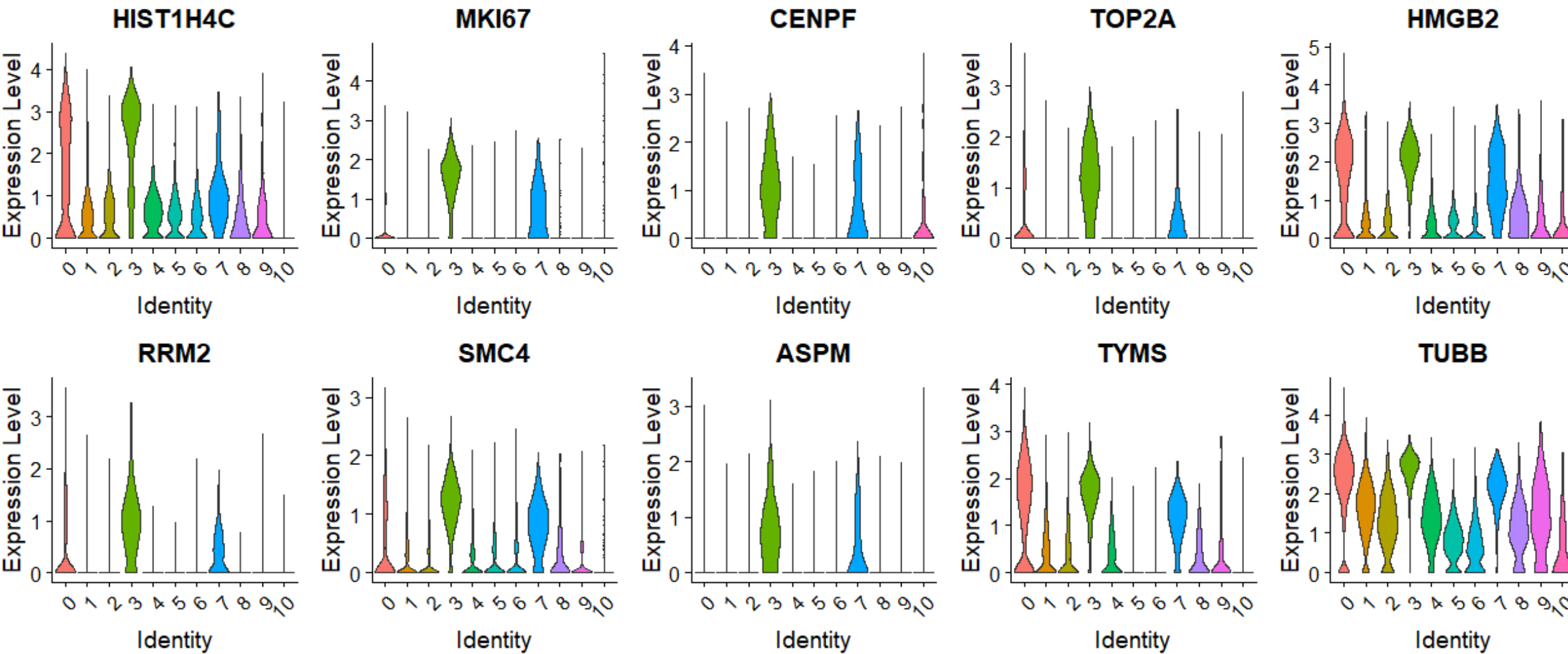

Cluster 3

Supplement: S10 Fig — (PDF) [file ppat.1010453.s010.pdf]

# Supplemental Figure 11

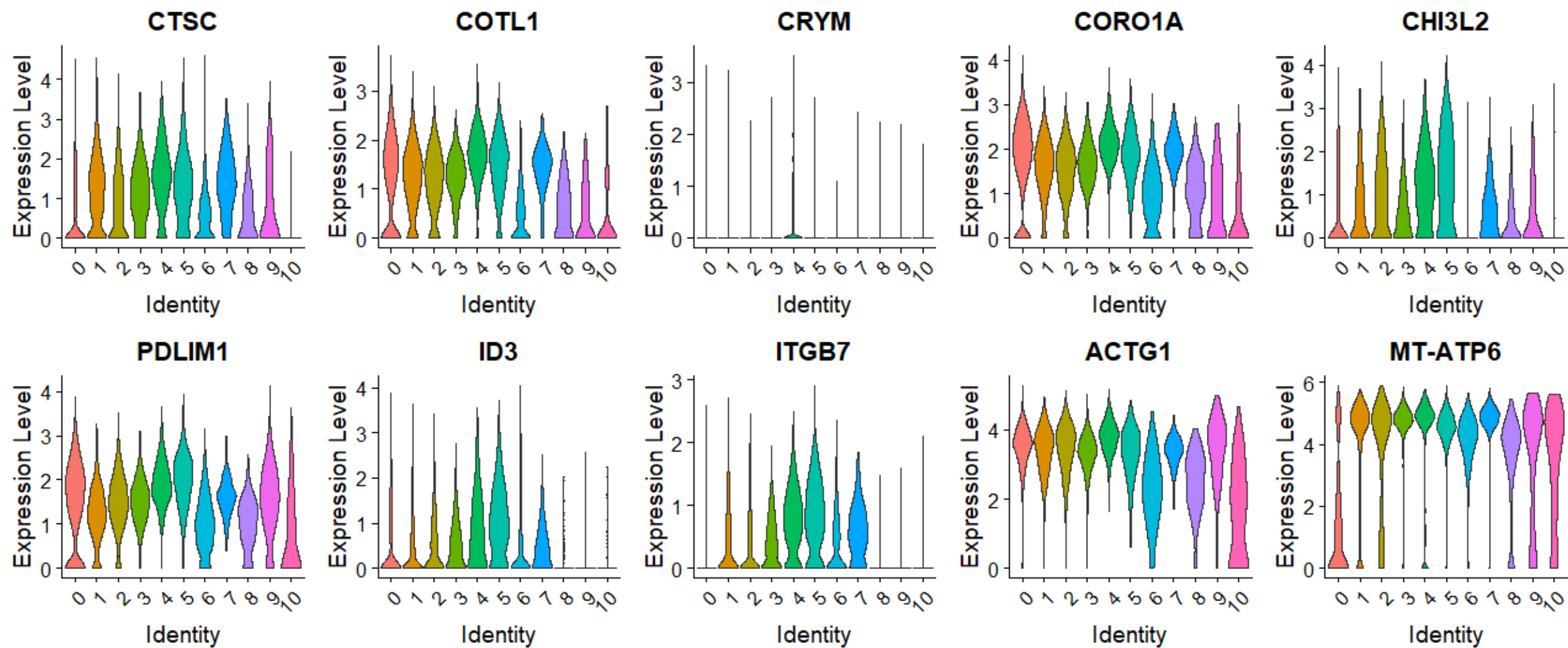

Cluster 4

Supplement: S11 Fig — (PDF) [file ppat.1010453.s011.pdf]

# Supplemental Figure 12

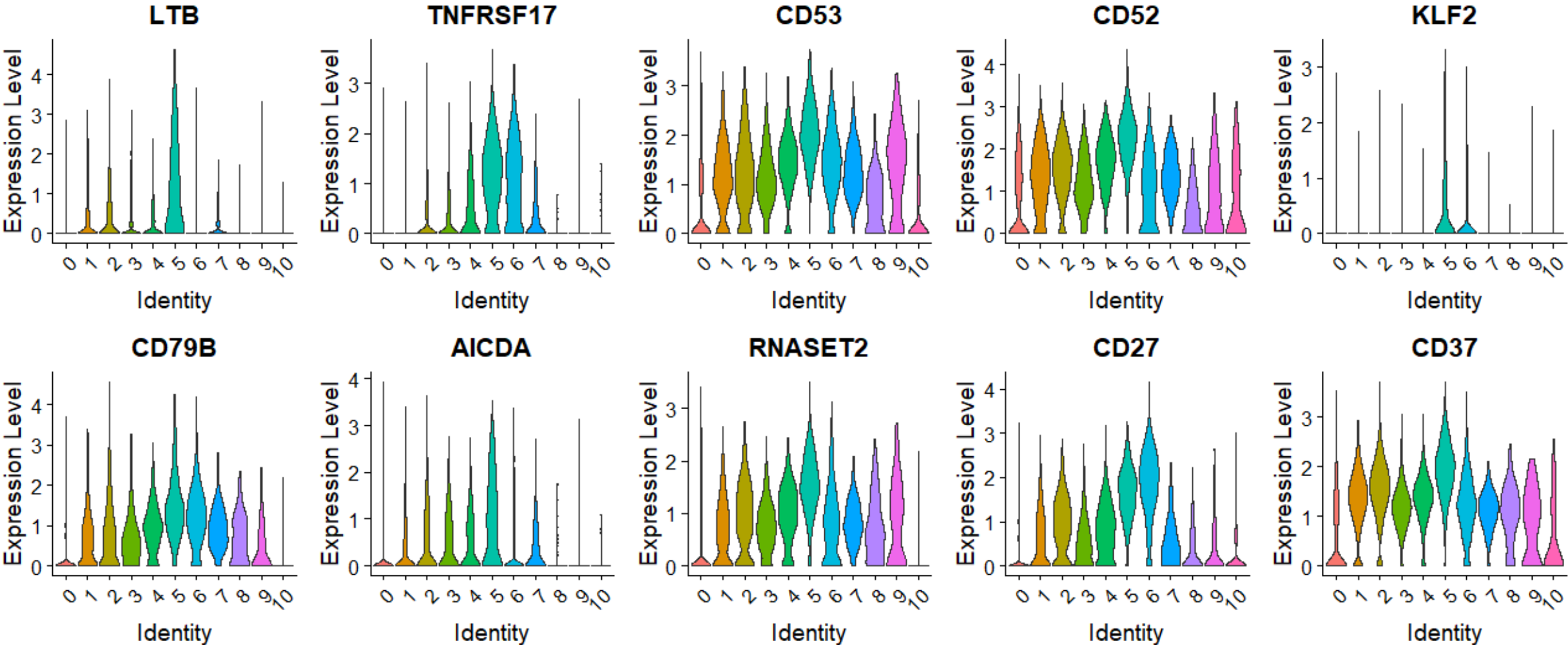

Cluster 5

Supplement: S12 Fig — (PDF) [file ppat.1010453.s012.pdf]

# Supplemental Figure 13

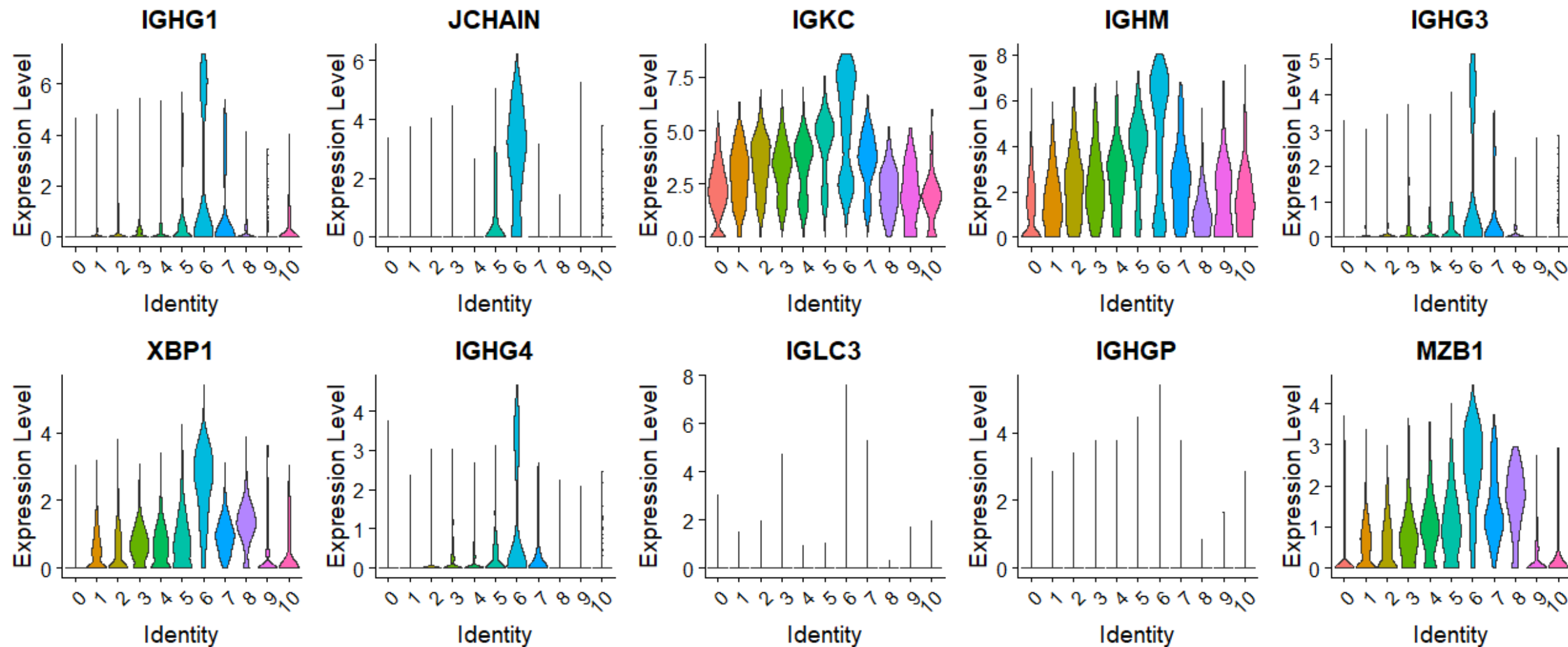

Cluster 6

Supplement: S13 Fig — (PDF) [file ppat.1010453.s013.pdf]

# Supplemental Figure 14

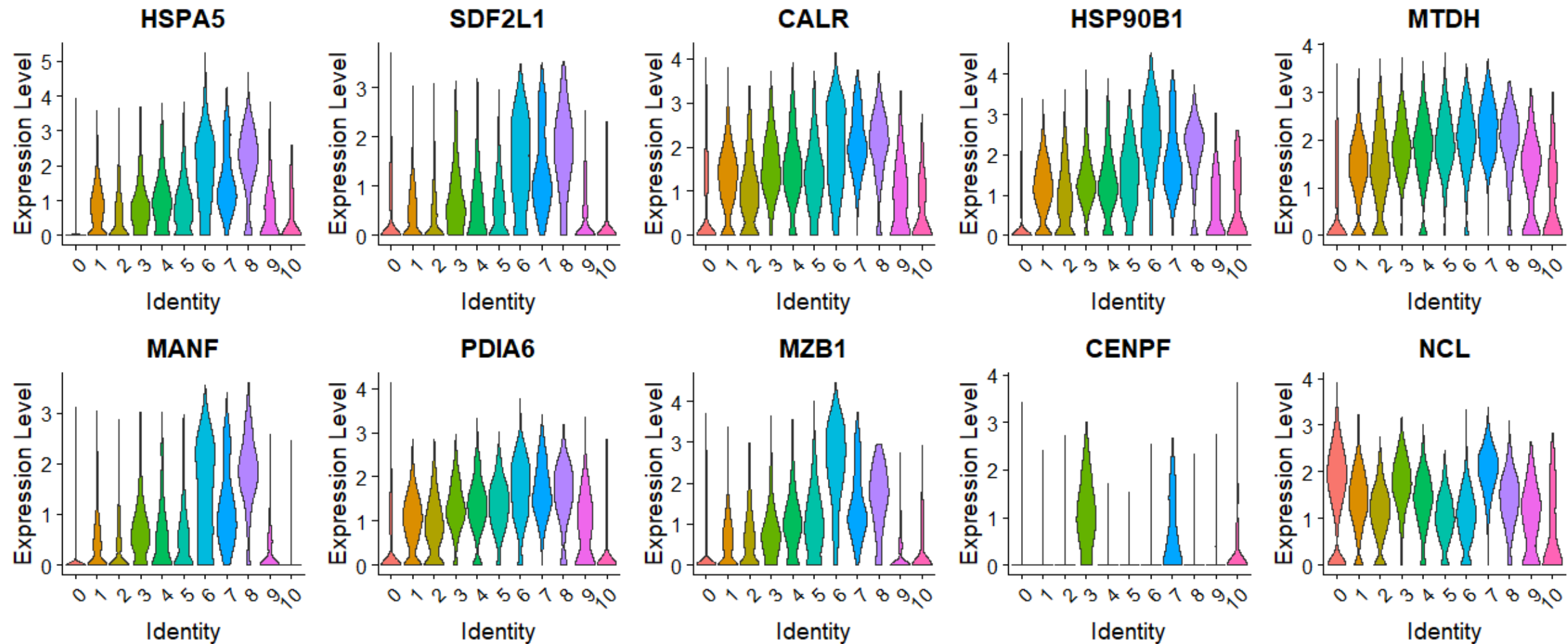

Cluster 7

Supplement: S14 Fig — (PDF) [file ppat.1010453.s014.pdf]

# Supplemental Figure 15

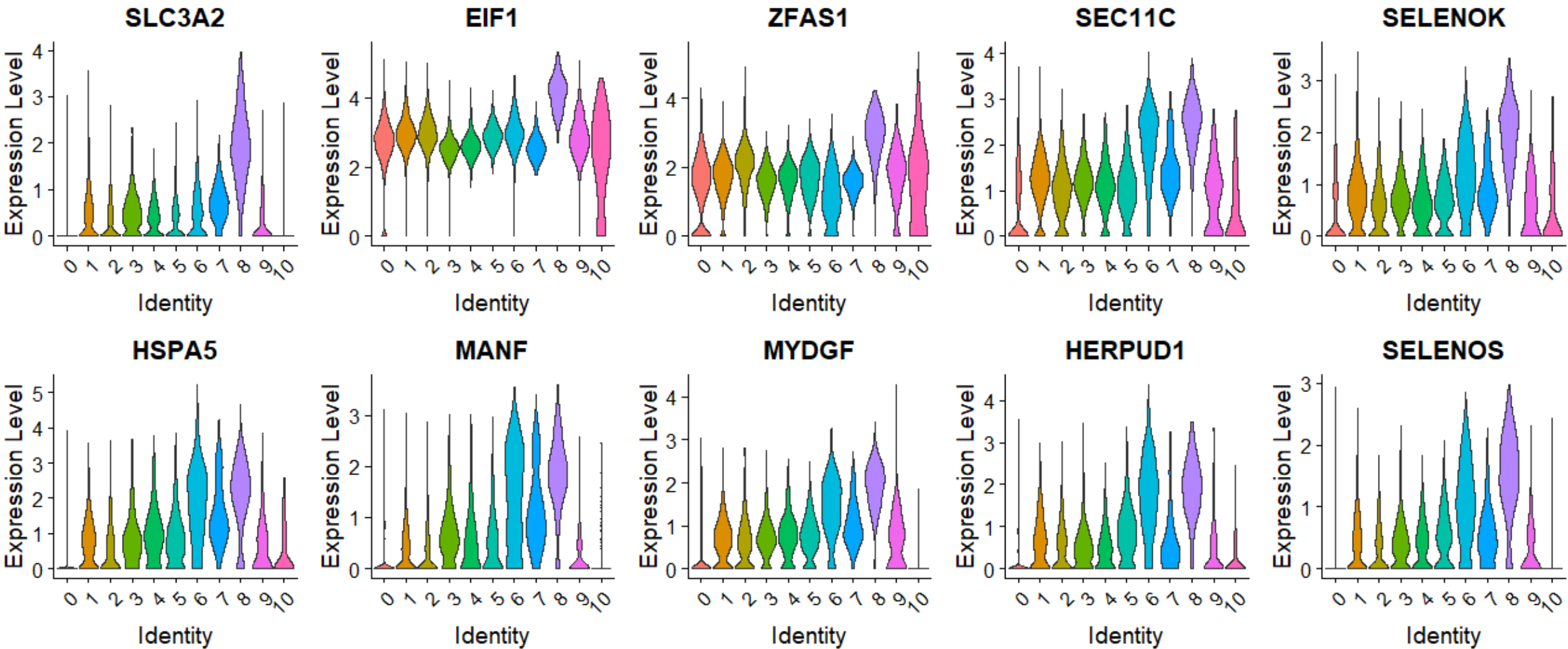

Cluster 8

Supplement: S15 Fig — (PDF) [file ppat.1010453.s015.pdf]

Supplemental Figure 16

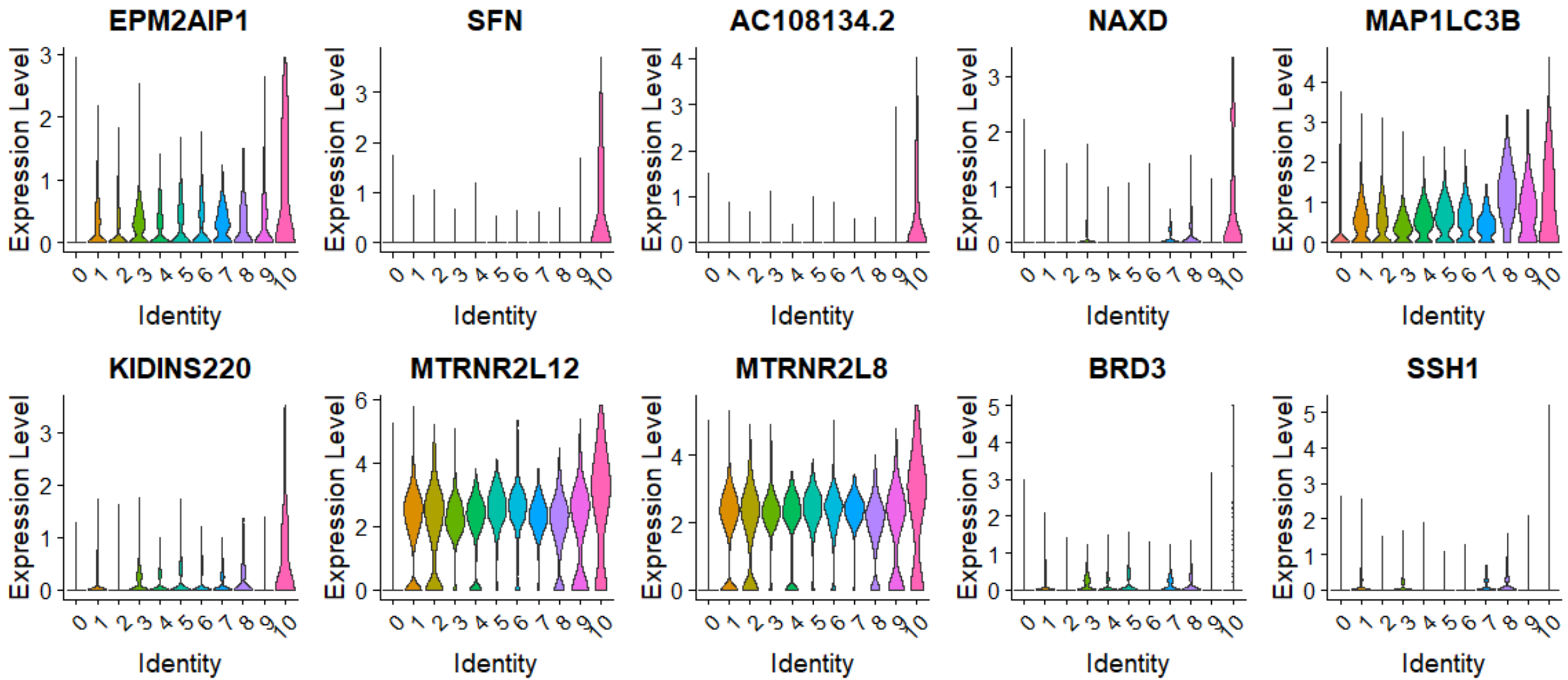

Cluster 10

Supplement: S16 Fig — (PDF) [file ppat.1010453.s016.pdf]

# Supplemental Figure 17

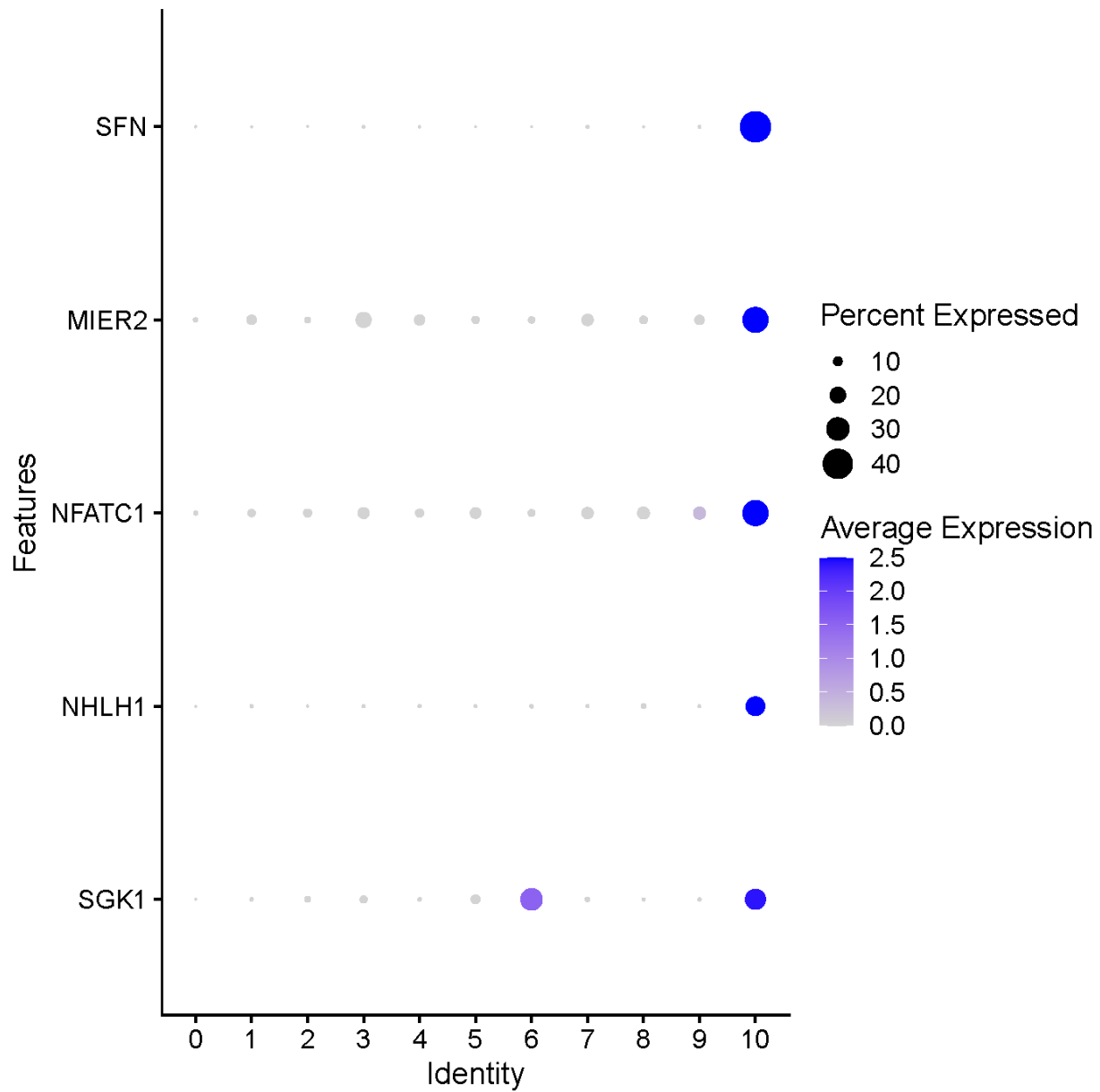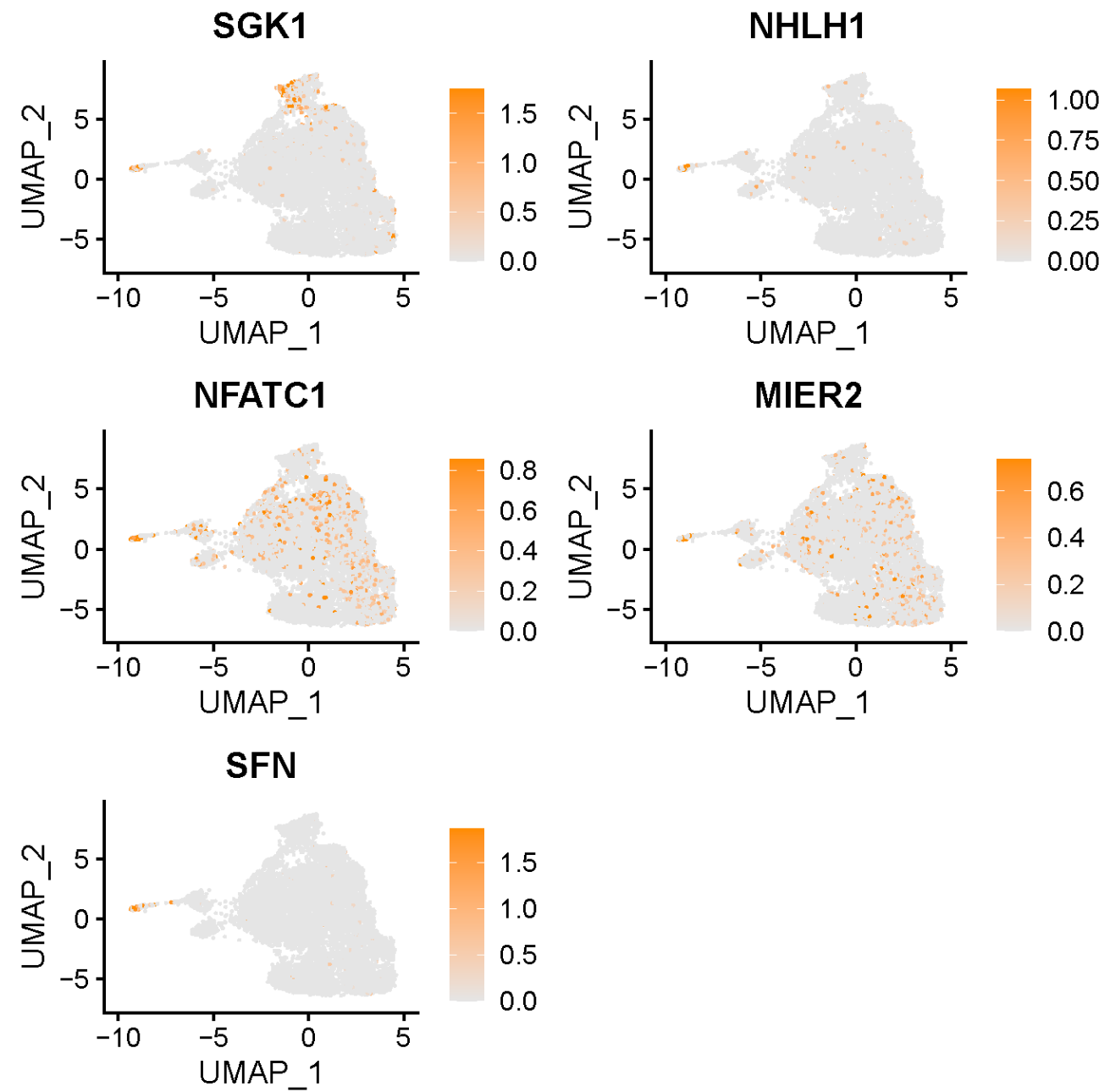

Supplement: S17 Fig — (PDF) [file ppat.1010453.s017.pdf]

# Supplemental Figure 18

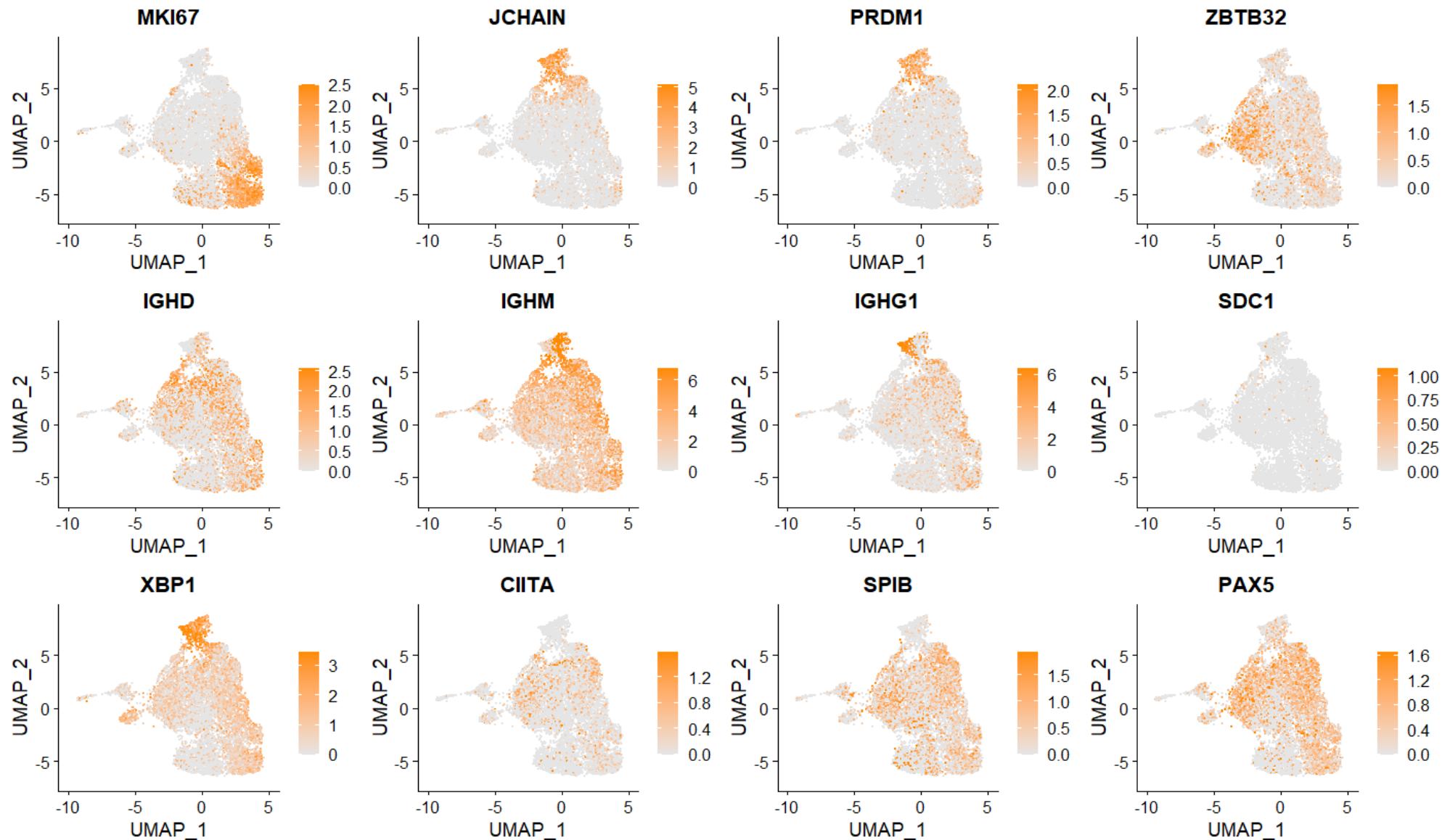

B cell/activation markers

Supplement: S18 Fig — (PDF) [file ppat.1010453.s018.pdf]
