# Supplementary material for: Reduced IRF4 expression promotes lytic phenotype in Type 2 EBV-infected B cells
Source: PLoS Pathog. 2022 Apr 26;18(4):e1010453. doi: 10.1371/journal.ppat.1010453 (PMC9041801; doi:10.1371/journal.ppat.1010453)
Supplement: S2 Table — Selected genes of interest that are upregulated in T2 LCLs compared to T1 LCLs in the RNA-seq results are shown, along with the fold-increase in gene expression and the adjusted p value. (DOCX) [file ppat.1010453.s020.docx]

| **Gene Symbol** | **log2-fold change** | **Adj. p** |
| --- | --- | --- |
| **NFATC1** | **0.8** | **0.05** |
| **Runx1** | **1.2** | **0.04** |
| **EGR1** | **1.4** | **0.03** |
| **ITGAX (CD11C)** | **3.2** | **0.01** |
| **ITGAM** | **1.9** | **0.03** |
| **GAS6** | **4.0** | **0.01** |
| **FYN** | **2.6** | **0.05** |
| **HCK** | **2.4** | **0.001** |
| **IL10** | **1.5** | **0.05** |
| **JAG1** | **2.2** | **0.01** |
| **CD9** | **1.7** | **0.01** |
| **CD5** | **2.2** | **0.03** |
| **CCR5** | **3.6** | **0.01** |
| **CCR2** | **3.0** | **0.05** |
| **CCRL2** | **2.0** | **0.02** |
| **TNFRSF9 (CD137)** | **1.7** | **0.04** |
| **CYP1B** | **3.1** | **0.01** |
| **TLR3** | **5.1** | **0.03** |
| **TLR4** | **3.2** | **0.02** |
| **WNT5A** | **4.8** | **0.04** |
| **PDGFRA** | **4.2** | **0.01** |
| **IL6R** | **2.5** | **0.05** |
| **IL9R** | **1.9** | **0.02** |
| **COL6A3** | **4.6** | **0.01** |
| **VCL** | **1.7** | **0.03** |
| **HLA-DRA** | **1.1** | **0.03** |
| **ENPP2** | **3.0** | **0.01** |

**S2 Table. Examples of Genes upregulated in T2 versus T1 lymphoblastoid cell lines.** Selected genes of interest that are upregulated in T2 LCLs compared to T1 LCLs in the RNA-seq results are shown, along with the fold-increase in gene expression and the adjusted p value.
