# Supplementary material for: Reduced IRF4 expression promotes lytic phenotype in Type 2 EBV-infected B cells
Source: PLoS Pathog. 2022 Apr 26;18(4):e1010453. doi: 10.1371/journal.ppat.1010453 (PMC9041801; doi:10.1371/journal.ppat.1010453)
Supplement: S3 Table — Selected genes of interest that are downregulated in T2 LCLs compared to T1 LCLs in the RNA-seq results are shown, along with the fold-increase in gene expression and the adjusted p value. (DOCX) [file ppat.1010453.s021.docx]

| **Gene Symbol** | **log2-fold change** | **Adj. p** |
| --- | --- | --- |
| **IRF4** | -0.4 | 0.03 |
| **EBF1** | -0.9 | 0.01 |
| **CASP1** | -2.7 | 0.01 |
| **GUCY1A1** | -5.0 | 0.01 |
| **GUCY1B1** | -3.7 | 0.03 |
| **PAG1** | -3.2 | 0.06 |
| **RGS1** | -3.3 | 0.01 |
| **MGAT5** | -4.2 | 0.01 |
| **GRAP2** | -7.4 | 0.01 |

**S3 Table. Examples of cellular genes down-regulated in T2 LCLs versus T1 LCLs.** Selected genes of interest that are downregulated in T2 LCLs compared to T1 LCLs in the RNA-seq results are shown, along with the fold-increase in gene expression and the adjusted p value.
