# Supplementary material for: Reduced IRF4 expression promotes lytic phenotype in Type 2 EBV-infected B cells
Source: PLoS Pathog. 2022 Apr 26;18(4):e1010453. doi: 10.1371/journal.ppat.1010453 (PMC9041801; doi:10.1371/journal.ppat.1010453)
Supplement: S4 Table — Selected genes of interest that are upregulated in IRF4-KO T1 LCLs and T2 LCLs are shown, along with the fold-increase in gene expression and the adjusted p value. (DOCX) [file ppat.1010453.s022.docx]

|  | **Control vs IRF4 KO LCL** | | **T2 vs T1 LCL** | |
| --- | --- | --- | --- | --- |
| **SYMBOL** | **log2-fold change** | **Adj. p** | **log2-fold change** | **Adj. p** |
| **NFATC1** | 0.7 | 6.89E-06 | 0.8 | 0.05 |
| **NFATC2** | 0.6 | 4.08E-05 | 1.8 | 0.10 |
| **ITGAX** | 1.0 | 1.46E-06 | 3.2 | 0.01 |
| **ITGAM** | 1.4 | 4.17E-11 | 1.9 | 0.03 |
| **FYN** | 1.1 | 3.89E-17 | 2.6 | 0.05 |
| **CD9** | 0.7 | 8.78E-05 | 1.7 | 0.01 |
| **JAG1** | 2.4 | 1.60E-26 | 2.2 | 0.01 |
| **CCND1** | 0.8 | 9.39E-05 | 2.1 | 0.02 |
| **TGFBR2** | 1.1 | 1.00E-03 | 2.7 | 0.03 |
| **FOXP4** | 0.7 | 1.77E-06 | 2.0 | 0.01 |
| **IL1R2** | 0.9 | 2.00E-04 | 2.5 | 0.02 |
| **PLAU** | 0.7 | 1.00E-03 | 2.5 | 0.01 |
| **TNFRSF9** | 1.3 | 6.37E-07 | 1.7 | 0.04 |
| **VCL** | 0.4 | 1.00E-02 | 1.7 | 0.03 |
| **GSN** | 1.3 | 8.04E-12 | 1.9 | 0.02 |

**S4 Table. Examples of Genes upregulated in both IRF4-KO (versus control) T1 LCLs and T2 (versus T1) LCLs.** Selected genes of interest that are upregulated in IRF4-KO T1 LCLs and T2 LCLs are shown, along with the fold-increase in gene expression and the adjusted p value.
